# A possible path to persistent re-entry waves at the outlet of the left pulmonary vein

**DOI:** 10.1038/s41540-024-00406-9

**Published:** 2024-07-23

**Authors:** Karoline Horgmo Jæger, Aslak Tveito

**Affiliations:** Department of Computational Physiology, Simula Research Laboratory, Oslo, Norway

**Keywords:** Biophysics, Computational biology and bioinformatics, Cardiology, Computer modelling, Dynamical systems

## Abstract

Atrial fibrillation (AF) is the most common form of cardiac arrhythmia, often evolving from paroxysmal episodes to persistent stages over an extended timeframe. While various factors contribute to this progression, the precise biophysical mechanisms driving it remain unclear. Here we explore how rapid firing of cardiomyocytes at the outlet of the pulmonary vein of the left atria can create a substrate for a persistent re-entry wave. This is grounded in a recently formulated mathematical model of the regulation of calcium ion channel density by intracellular calcium concentration. According to the model, the number of calcium channels is controlled by the intracellular calcium concentration. In particular, if the concentration increases above a certain target level, the calcium current is weakened to restore the target level of calcium. During rapid pacing, the intracellular calcium concentration of the cardiomyocytes increases leading to a substantial reduction of the calcium current across the membrane of the myocytes, which again reduces the action potential duration. In a spatially resolved cell-based model of the outlet of the pulmonary vein of the left atria, we show that the reduced action potential duration can lead to re-entry. Initiated by rapid pacing, often stemming from paroxysmal AF episodes lasting several days, the reduction in calcium current is a critical factor. Our findings illustrate how such episodes can foster a conducive environment for persistent AF through electrical remodeling, characterized by diminished calcium currents. This underscores the importance of promptly addressing early AF episodes to prevent their progression to chronic stages.

## Introduction

Atrial fibrillation (AF) is the most common cardiac arrhythmia, characterized by rapid and irregular beating of the atria. This can lead to symptoms like palpitations, fatigue, and shortness of breath, increasing the risk of stroke and heart failure^1,2^, along with a series of other possible health issues^3^. The prevalence of AF is increasing and it is expected to affect more than 8 million people in the USA by 2050, and more than 18 million in the EU by 2060^4^. Indeed, significant societal and economic impacts of long-term AF are well-documented^2,3,5^. While the biophysical mechanisms underlying AF have been studied for over a century, their precise nature remains a subject of ongoing debate^6–11^. Understanding of the mechanisms is crucial for effective treatment, including both ablation techniques and pharmacological interventions.

It appears to be a widespread consensus that AF typically progresses from paroxysmal episodes to persistent and eventually permanent forms^10,12^. In this report, we explore a hypothesis aiming to explain the progression from paroxysmal to permanent AF. Utilizing a recently developed mathematical model, we consider the potential impact of high-frequency firing over a limited period on the permanent establishment of re-entry, driven by remodeling of the calcium current in the cell membrane. The model suggests that increased intracellular calcium concentrations, triggered by high-frequency firing, lead to a reduction in the calcium current. This, in turn, shortens the action potential duration, thereby increasing the likelihood of re-entry. We present computational evidence to support the plausibility of this hypothesis, particularly focusing on the dynamics at the outlet of the left pulmonary vein (PV).

The significance of the pulmonary veins’ outlets as primary sources of AF is well-established in the literature^13,14^. The prevalence of ectopic beats originating from this region is also well-documented, see, e.g.^15^. It is a recognized phenomenon that the rapid firing of action potentials in the atria leads to an increase in intracellular calcium concentration^16^. Furthermore, there is evidence suggesting that the calcium current is reduced during such rapid pacing^11,17–19^, which in turn, is associated with a decrease in the duration of the action potential^20^.

**Fig. 1:**
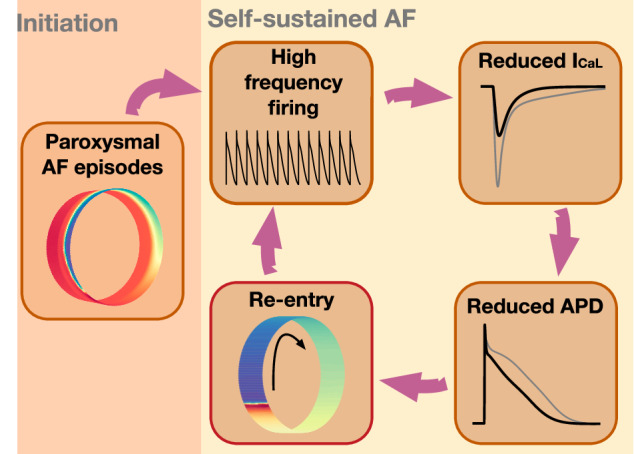
Hypothesis: the vicious cycle. Illustration of the proposed path to sustained re-entry around the PV sleeve. Paroxysmal AF episodes in the PV sleeve leads to a high frequency of action potential firing. According to the model (10), this leads to a reduction in the number of L-type calcium channels in the membrane of the cardiomyocytes in order to compensate for the increased intracellular calcium concentration resulting from rapid firing. The reduction in the number of L-type calcium channels reduces the L-type calcium current, *I*_CaL_. This reduction results in a reduction in the action potential duration (APD), which increases the likelihood of a re-entrant wave being able to travel around the PV sleeve. Such a re-entrant wave in itself results in a high firing frequency for the cardiomyocytes around the PV sleeve, which maintains the reduced *I*_CaL_ and reduced APD, promoting the continued existence of the re-entrant activity.

When these observations are synthesized, the hypothesis presented above (see also Fig. 1) is neither novel nor unexpected. However, the primary aim of this report is to demonstrate how these disparate elements coalesce within a computational model, thereby reinforcing the plausibility of the hypothesis.

We will use a recently developed mathematical model to demonstrate a possible progressive path to sustained re-entry. In a series of papers, a theory on the regulation of the density of ion channels has been developed, see refs. ^21–28^. For the regulation of the calcium current, the essence of this theory is that the density of ion channels is governed by the intracellular calcium concentration. If the intracellular calcium concentration, *c*, falls below a target value, c^*^, the number of channels responsible for the *I*_CaL_-current is increased. The upregulation of the calcium current, *I*_CaL_, leads to a larger influx of Ca^2+^ into the cell and, as a result, more Ca^2+^ is released from internal storage systems because of the process referred to as graded release, see, e.g.^29–31^. The upregulation of *I*_CaL_ channels is continued until equilibrium is reached, i.e., until *c* = c^*^. Conversely, if *c* > c^*^, the number of ion channels is reduced until *c* is reduced to the target value. This model was developed for neurons^21–24^, and applied to study the dynamics of the Sino-atrial node^32^. Recently, the model was used to explain the time-dependent efficacy of calcium channel blockers^28^. Note that, for instance, in refs. ^24,32^ other currents and fluxes were also regulated by the level of intracellular calcium concentration, but we will concentrate on the *I*_CaL_ current for reasons that are explained in the Discussion.

The hallmark of AF is an extremely high beat rate in the upper heart. This high-frequency beating subsequently leads to increased *c* and this, in turn, according to the theory referred to above, down-regulates the *I*_CaL_-current. Reduced *I*_CaL_-current results in reduced action potential duration (APD) and this can be critical for maintaining a re-entry wave. We will specifically show that sustained re-entry of an excitation wave around the PV sleeve can be achieved in the following sequence 1) fast pacing by paroxysmal AF episodes, 2) down-regulation of the *I*_CaL_-current, 3) reduced APD, 4) high-frequency pacing is self-sustained by the stable re-entry; adding up to a vicious cycle, see Fig. 1.

## Results

In this section, we present results of numerical simulations. First, we confirm the assumption that a reduction in *I*_CaL_ promotes the existence of a re-entrant wave around the computational pulmonary vein sleeve. Next, we confirm that rapid pacing resulting from paroxysmal AF leads to a reduction in *I*_CaL_ in left atrial (LA) and pulmonary vein (PV) cardiomyocyte membrane models with a dynamic number of *I*_CaL_ channels. This reduction in *I*_CaL_ is next shown to, in some cases, be sufficient for a re-entrant wave around the PV sleeve to be generated. Such a re-entrant wave would then in itself be responsible for rapid pacing and a reduced *I*_CaL_, making the mechanism self-sustained (see Fig. 1). Finally, we show how perturbations of physiological parameters influence the forming of re-entry waves.

### Reduced *I*_CaL_ promotes re-entry around the PV sleeve

**Fig. 2:**
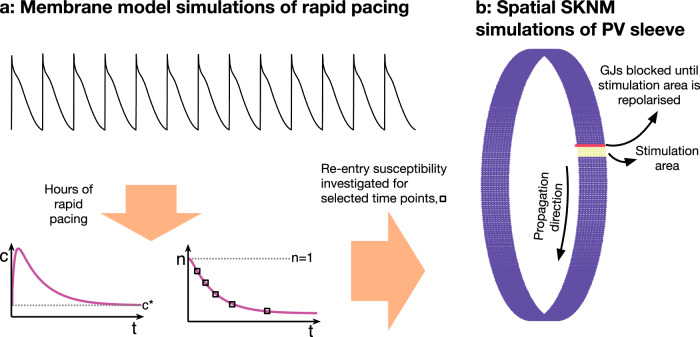
Computational procedure. Illustration of the computational procedure used to investigate re-entry susceptibility in the PV sleeve. **a** First, membrane model simulations of the LA and PV cardiomyocytes with rapid pacing are conducted. After minutes and hours of rapid pacing, the number of *I*_CaL_ channels, *n*, is considerably reduced to compensate for the increased intracellular calcium concentration, *c*, resulting from the rapid pacing. **b** At some selected points in time, *n* is collected from the membrane model simulations and a spatial simulation is conducted to investigate if a re-entrant wave traveling around the PV sleeve can be generated. In order to generate a wave traveling in one direction, the cells in a small area of the PV sleeve are stimulated and the gap junction (GJ) connections on the boundary of this area in one of the directions are blocked until the stimulated cells are repolarized.

**Fig. 3:**
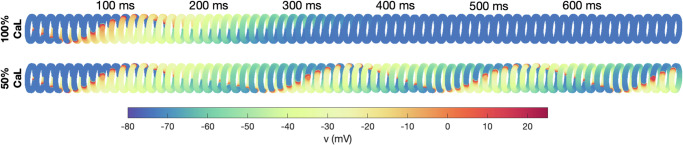
Re-entry around the PV sleeve for reduced *I*_CaL_. The figure shows the membrane potential for the cells in the PV sleeve cylinder at some different points in time after a stimulation is applied (see Fig. 2b), representing an ectopic beat. The upper panel shows the solution in the default case, and the lower panel shows the solution when the *I*_CaL_-current has been reduced by 50%, which induces sustained re-entry.

In order to investigate re-entry susceptibility, we use the same set-up as in ref. ^33^, illustrated in Fig. 2b. The upper panel of Fig. 3 shows the solution of such a simulation in the default case. We observe that following a stimulation (e.g., an ectopic beat), an excitation wave is generated traveling around the PV sleeve, but when the wave has traveled one round around the cylinder, the initially stimulated cells are not sufficiently repolarized to be excited again and the wave terminates after traveling one round.

In the lower panel of Fig. 3, however, we consider the case of *I*_CaL_ reduced by 50%. In this case, the cells repolarize faster (shorter APD) and the initially stimulated cells are ready to be excited again when the excitation wave has traveled one round. Consequently, the wave continues to continuously travel around and around the PV sleeve. The wave travels with a conduction velocity of about 29.6 cm/s. This means that the wave uses about 160 ms to travel around the cylinder of diameter 1.5 cm, corresponding to a beat rate of about 6.3 beats per second (bps) or 375 beats per minute (bpm) for the PV sleeve cardiomyocytes.

### A computational path to sustained re-entry

Figure 3 illustrates that a reduction in the *I*_CaL_-current might promote the generation of a re-entrant wave around the PV sleeve resulting in a high beat rate. The purpose of this paper is to illustrate that such a reduction in the *I*_CaL_-current might result from rapid firing because rapid firing increases the intracellular calcium concentration, which according to the model (10) would result in a reduction in the number of *I*_CaL_-channels, *n*. As illustrated in Fig. 1, once a re-entrant wave is generated, this re-entrant wave would result in rapid firing of the cells which could maintain the reduced number of *I*_CaL_ channels and increased re-entry susceptibility in a self-sustained manner. However, in order for the re-entrant wave to be generated in the first place, some other mechanism (e.g., paroxysmal AF episodes, see Fig. 1) is needed to produce the initial rapid beating resulting in the initial reduction of the number of *I*_CaL_-channels.

We investigate this mechanism using the set-up described in Fig. 2. First, we perform membrane model simulations of rapid pacing and observe how the number of *I*_CaL_-channels, *n*, is reduced over time. For a number of selected time points, we then investigate whether the *I*_CaL_ reduction at that point in time is enough for a re-entrant wave to be generated using a spatial representation of the PV sleeve. We define a re-entrant wave to be generated if any of the PV sleeve cells are depolarized after 500 ms (see Fig. 3).

### Development of the *I*_CaL_ current under influence of rapid-firing

**Fig. 4:**
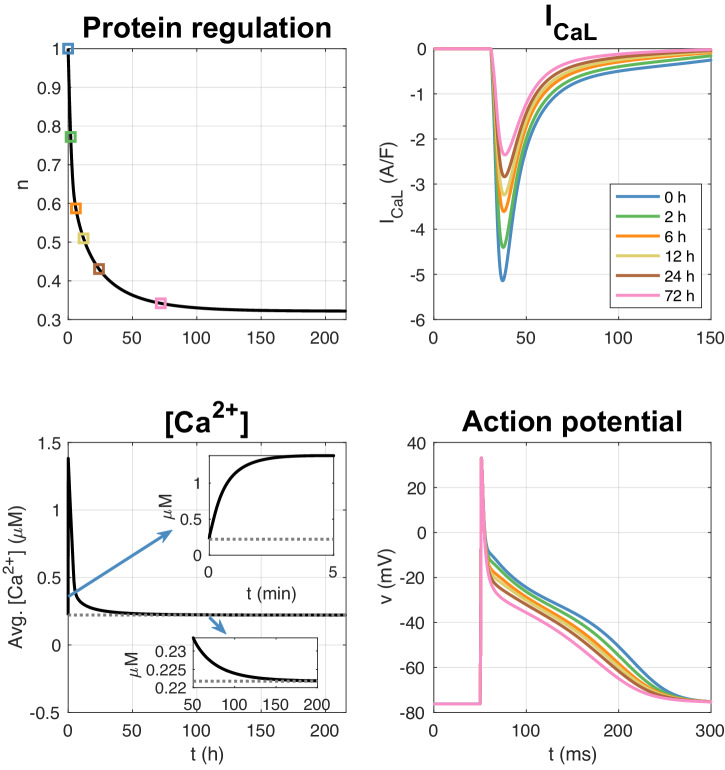
LA membrane model simulation with pacing of 10 bps. The upper left panel shows how the relative number of *I*_CaL_ channels, *n*, is reduced in response to the increased intracellular calcium concentration resulting from the rapid pacing. The lower left panel shows the average intracellular calcium concentration (averaged over a second) as a function of time. The gray dotted line is the target calcium concentration, c^*^. The upper right panel shows *I*_CaL_ at given points in time during the simulation, and the lower right panel shows the corresponding action potentials.

In Fig. 4, we show the solution of an LA membrane model simulation with a pacing of 10 bps (or 600 bpm). We observe that the intracellular calcium concentration increases as a result of the rapid pacing and that the number of *I*_CaL_ channels consequently is reduced. After about 12 h, the number of *I*_CaL_ channels is reduced to about 50% (*n* = 0.5) and after about 150 h, the number of channels is reduced to about 32% (*n* = 0.32). The right panel illustrates *I*_CaL_ and the action potential as some selected points in time during the simulation. We observe that the action potential duration is reduced as the rapid pacing continues.

**Fig. 5:**
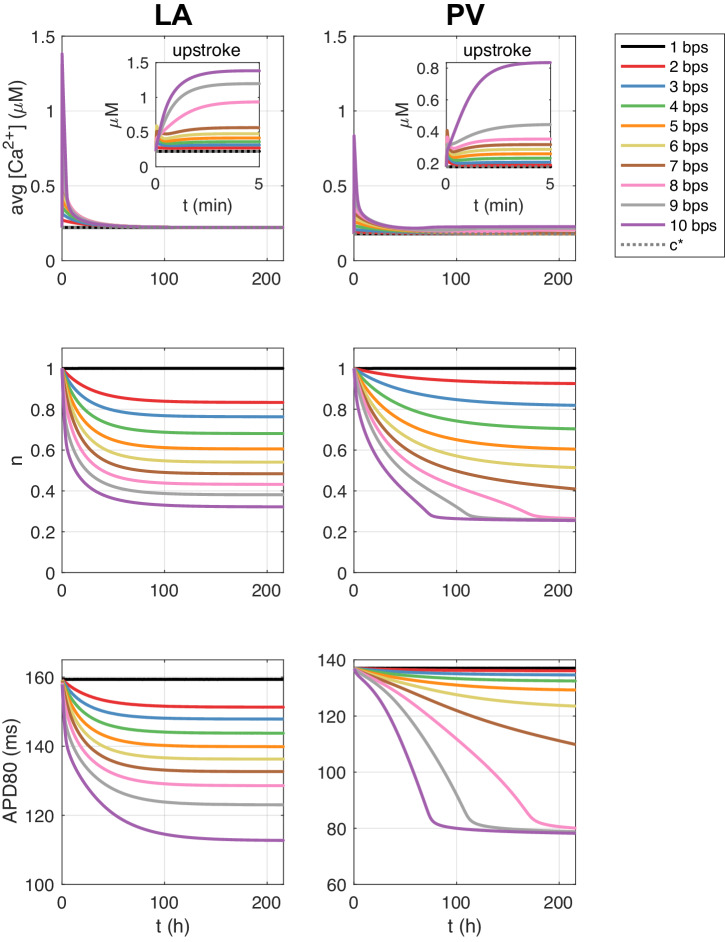
Membrane model simulations with rapid pacing. Effect of different pacing frequencies on the average (over one second) intracellular calcium concentration, the relative number of *I*_CaL_ channels, *n*, and the action potential duration at 80% repolarization (APD80) in membrane model simulations of the LA and PV models.

In Fig. 5, we show the solution of similar simulations using different pacing frequencies for both the LA and PV membrane models. We observe that the reduction in the number of *I*_CaL_ channels and the reduction in the action potential duration is faster and more pronounced as the pacing frequency increases.

### Increased re-entry susceptibility resulting from long-term rapid pacing

**Fig. 6:**
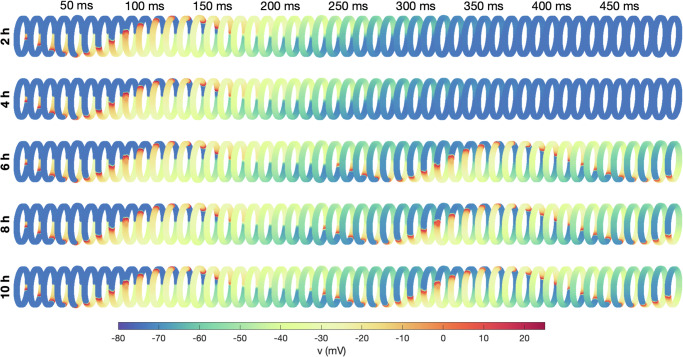
PV sleeve simulations after 2 h, 4 h, 6 h, 8 h, and 10 h of rapid (10 bps) pacing. The figure shows the membrane potential for the cells in the PV sleeve cylinder at some different points in time after a stimulation is applied (indicated in the top text). Each row corresponds to a time point in the membrane model simulations of rapid pacing described in the text to the right. The simulation set-up is illustrated in Fig. 2.

In Fig. 6, we have plotted the membrane potential of the cells in spatial simulations of the PV sleeve (see Fig. 2b). The value of the relative number of *I*_CaL_ channels, *n*, for the PV and LA cells are collected from different points in time in membrane model simulations with pacing of 10 bps. We observe that after 2 h and 4 h of rapid pacing, the number of *I*_CaL_ channels is not reduced enough for a re-entrant wave to be generated, but after 6 h (and 8 h and 10 h) a re-entrant wave is generated.

**Fig. 7:**
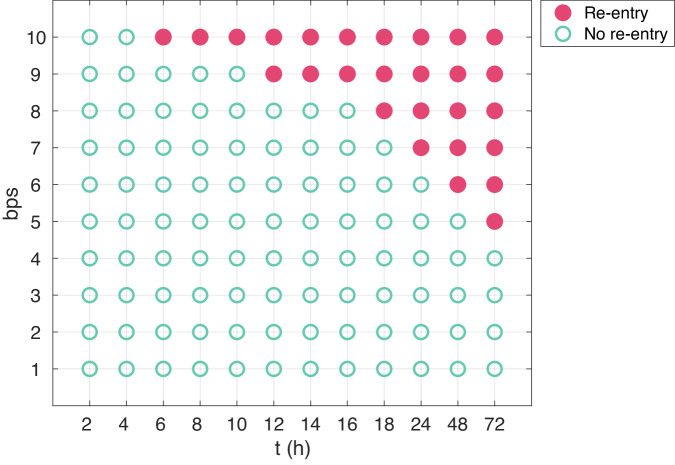
Re-entry susceptibility after hours of rapid pacing. The figure shows an overview of whether or not a re-entrant wave is generated after hours of different degrees of rapid pacing. Membrane model simulations are used to find the relative number of *I*_CaL_ channels, *n*, at the different points in time (see Fig. 2a), and spatial simulations of the PV sleeve is used to investigate whether a re-entrant wave is generated (see Fig. 2b).

In Fig. 7, we have performed the same type of simulation for a number of different time points and pacing frequencies and report whether or not a re-entrant wave is generated. We observe that the probability of re-entry increases as the number of beats per second increases and also when the duration of rapid pacing is increased.

### Influence of parameter perturbations

**Fig. 8:**
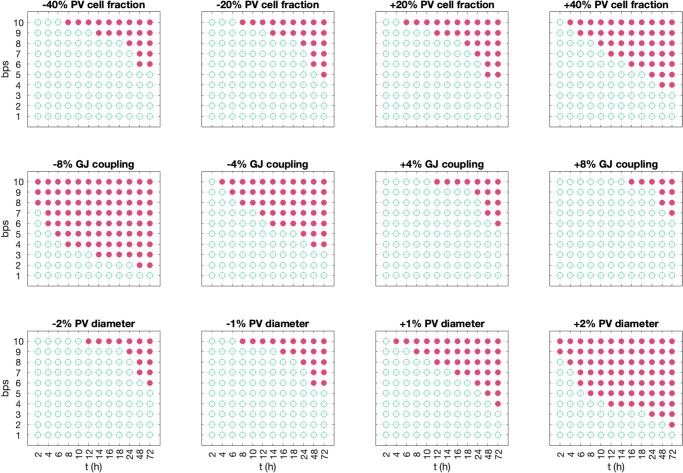
Re-entry susceptibility after hours of rapid pacing for variations of parameter values. The figure shows an overview of whether or not a re-entrant wave is generated after hours of different degrees of rapid pacing using the set-up described in Fig. 2 for a number of adjusted parameter values. A closed red circle indicates that a re-entrant wave was generated, and an open green circle indicates that a re-entrant wave was not generated. In the upper panel, we have adjusted the fraction of PV myocytes in the PV sleeve from the default fraction of 50% PV myocytes and 50% LA myocytes. In the middle panel, we have adjusted the default strength of the gap junction coupling, ${G}_{g}^{* ,l}=\frac{1}{{R}_{g}^{* ,l}}$ and ${G}_{g}^{* ,t}=\frac{1}{{R}_{g}^{* ,t}}$, where ${R}_{g}^{* ,l}$ and ${R}_{g}^{* ,t}$ are given in Table 1. In the lower panel, we have varied the diameter of the PV sleeve by increasing or decreasing the number of surrounding cells.

**Fig. 9:**
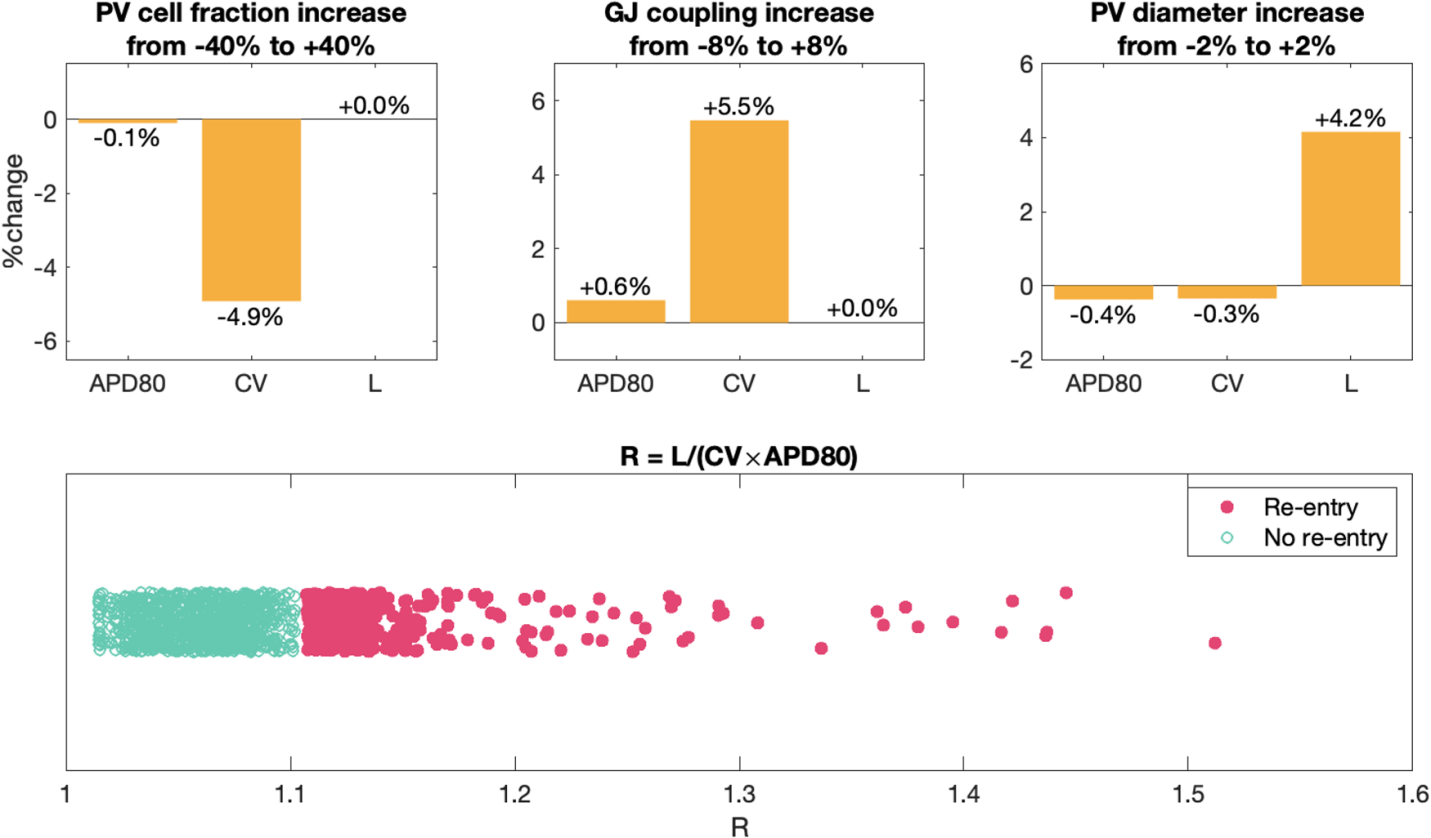
Effect of the parameter perturbations considered in Fig. 8 on relevant biomarkers. In the upper panel, we report the average change in action potential duration (APD80), conduction velocity (CV) and PV sleeve circumference (L) resulting from increasing the model parameters from the smallest to the largest values considered in Fig. 8. For example, in the upper left panel we observe that when the PV cell fraction increases from 40% below the baseline value to 40% above the baseline value, APD80 is almost unchanged (decreases by 0.1%), whereas the CV decreases by 4.9%. The PV sleeve circumference, L, is not affected by the PV cell fraction. Similarly, increasing the gap junction coupling from 8% below baseline to 8% above baseline increases the CV by 5.5%. In addition, increasing the PV diameter directly increases the circumference, L. In the lower panel, we show the value of R = L/(CV × APD80) for each of the simulations in Fig. 8 and mark whether or not a re-entrant wave was generated. The definitions used to compute APD80, CV and L are described below.

In Fig. 8, we investigate how the results in Fig. 7 depend on perturbations of essential model parameters. In addition, Fig. 9 shows changes to the conduction velocity (CV), the action potential duration (APD80), and the circumference of the PV sleeve (L) resulting from these parameter perturbations.

#### Changing PV/LA-distribution

In the upper panel of Fig. 8, we vary the fraction of PV myocytes in the PV sleeve, from the default fraction of 50% PV myocytes and 50% LA myocytes. We observe that as the fraction of PV myocytes increases, less time of rapid pacing and less frequent pacing is required before a re-entrant wave is generated. In Fig. 9, we observe that increasing the fraction of PV cells results in a reduced CV. This is likely the cause of the increased re-entry susceptibility associated with an increased PV cell fraction (see Discussion).

#### Changing the gap junction density

Similarly, in the middle panel of Fig. 8, we vary the strength of the default gap junction coupling between myocytes. We observe that if the gap junction coupling is, e.g., reduced by 8%, considerably less time of rapid pacing is required before to generate re-entry around the PV cylinder and considerably less frequent pacing is required. Conversely, if the gap junction coupling is increased by 8%, more frequent and long-lasting pacing is required to generate re-entry. In Fig. 9, we observe that increasing the gap junction coupling results in an increased CV, which is likely the cause of the observed reduced re-entry susceptibility associated with gap junction coupling increase (see Discussion).

#### Changing the diameter of the pulmonary vein

Finally, in the lower panel of Fig. 8, we vary the diameter of the PV sleeve by increasing of decreasing the number of surrounding myocytes. In this case, increasing the PV sleeve diameter considerably reduces the frequency and duration of rapid pacing required to induce a re-entrant wave around the PV sleeve.

## Discussion

It is well-established that atrial fibrillation (AF) follows the concept of *AF begets AF*, signifying its progressive and self-perpetuating nature, see, e.g.,^34–37^. This implies that episodes of AF contribute to the development of a conducive substrate for subsequent occurrences, thereby rendering the condition progressively more entrenched and potentially leading to a self-sustaining mechanism. Here, we have proposed a pathway from paroxysmal AF episodes to sustained re-entry at the left pulmonary vein’s outlet, employing a mathematical model to expose this progression, see Fig. 1.

In Fig. 3, we show how excitation waves can propagate around the pulmonary vein outlet. We examine two scenarios: a control case and one where the calcium current, *I*_CaL_, is halved. A reduction in *I*_CaL_ is associated with a decrease in action potential duration (APD), which, as demonstrated in the simulation depicted in Fig. 3, leads to a heightened risk of re-entry wave formation. The relationship between diminished calcium current and shortened APD has been well-documented, see refs. ^38,39^. This, in turn, results in a reduced effective refractory period, escalating the likelihood of re-entrant wave emergence.

The effect of rapid firing on the strength of the calcium current as modeled by (10) is shown in Fig. 4. Rapid firing leads to increased cytosolic calcium load which according to the model (10) reduces the calcium current. The effect of increased cytosolic calcium load during rapid firing is well documented^39^ and easily seen in simulations based on AP models of atrial cells, see Supplementary Note 1. Weakened calcium current during rapid firing is also well documented, see, e.g.,^17,18,39–41^. Here we suggest that the remodeling can be represented by (10). According to this model, the calcium current, *I*_CaL_, is down-regulated when the cytosolic calcium concentration is elevated above its target value.

In Fig. 6, we demonstrate that re-entry may develop after extended periods of rapid firing. For this example, we simulated the membrane model (no spatial variation) continuously for up to ten hours to monitor the impact of sustained rapid firing on the calcium current dynamics. At intervals of 2 h, 4 h, 6 h, 8 h, and 10 h, we analyzed the spatial model’s behavior. It was noted that at the 2 h and 4 h marks, the excitation waves ceased after a single circumnavigation of the pulmonary vein. However, with prolonged rapid firing – specifically after 6 h, 8 h, and 10 h – a consistent re-entry pattern emerged. This pattern suggests that the cellular remodeling induced by the rapid firing persists for several hours before the re-entry stabilizes. In Fig. 7, we show how the path to re-entry depends on the frequency and duration of the firing. More prolonged, and faster, rapid firing clearly increases the occurrence of re-entry.

Figure 8 illustrates how alterations in tissue properties significantly influence the propensity for re-entry. As noted above, the ratio of pulmonary vein (PV) to left atrium (LA) myocytes critically affects the likelihood of re-entry events. An increased proportion of PV myocytes correlates with a higher incidence of re-entry. Likewise, diminished gap junction (GJ) coupling is associated with more frequent re-entry occurrences. The diameter of the pulmonary vein plays a crucial role; even a slight enlargement significantly raises the incidence of re-entry.

The mechanisms for these effects relate to the time it takes for an excitation wave to travel around the PV geometry compared to the duration of the action potential, APD. The travel time, T, equals the circumference (L) of the PV divided by the conduction velocity (CV); thus, ${{{\rm{T}}}}=\frac{{{{\rm{L}}}}}{{{{\rm{CV}}}}}$. Re-entry requires APD to be sufficiently smaller than T since the cells must be prepared to be re-excited when the electrical front completes its circuit. Specifically, by defining the ratio

$${{{\rm{R}}}}=\frac{{{{\rm{L}}}}}{{{{\rm{APD}}}}\times {{{\rm{CV}}}}}$$

we expect that the likelihood of re-entry will increase as R increases. In Fig. 8, we show how distributions of PV/LA myocytes, perturbations of the perimeter of the pulmonary vein, and the strength of the gap junction coupling affects the propensity for re-entry for different beat rates. The data from this figure is used to compute the factor R as shown in the lower panel of Fig. 9 where we notice that as R increases, so does the likelihood of re-entry. To summarize, the probability of re-entry increases if APD is reduced, which occurs when the calcium current is enhanced; it rises if the circumference L of the PV is increased, and it increases if CV is decreased as happens if the gap junction coupling is weakened or the proportion of PV cells is increased.

In the models presented in refs. ^24,32^, regulation by cytosolic calcium concentration extends beyond the calcium current. In ref. ^24^ regulation of all membrane currents was included, while ref. ^32^ in addition allowed aspects of intracellular calcium dynamics to be controlled by the calcium concentration. Although our primary focus remains on the calcium current, as in ref. ^28^, we acknowledge that other currents and fluxes may also be impacted. In Supplementary Note 1, we show the effect of increasing the individual membrane currents by 20%. As seen in Supplementary Fig. 2, increase in the *I*_CaL_ current, increases the intracellular calcium concentration. Therefore, regulation of calcium overload by decreasing the number of calcium channels is reasonable. The same effect holds for the background calcium current. But the fast sodium current *I*_Na_ in both LA and PV cardiomyocytes have very limited effects on the intracellular calcium concentrations. Therefore, regulation of this current in order to repair deviation from the calcium target value appears to be ineffectual. Several of the potassium currents work the other way around compared to *I*_CaL_ and cannot be used to control intracellular calcium in the same way as the calcium current. A comprehensive review of cardiac ion channel remodeling is given in ref. ^41^. For atrial cardiomyocytes, *I*_CaL_ is weakened during AF, but the potassium currents have more complex behavior. For instance, the *I*_to_ current (transient outward potassium current) is decreased, but the *I*_K1_ current (inward rectifier potassium current) is increased. To conclude, it is not straightforward to use the model (10) for all the currents of the atrial cell membrane in the way suggested by refs. ^24,32^, and we therefore restrict our attention to the *I*_CaL_ current. These considerations and the conclusion are in line with the discussion of similar issues in ref. ^28^.

Although we only alter the density of channel proteins for the *I*_CaL_ current, leaving the density of other channels, pumps and exchangers unchanged, the changes in the intracellular ion concentrations and the number of *I*_CaL_ channels following rapid pacing clearly affect the currents through other ion channels, pumps and exchangers during an action potential; see Supplementary Note 2. Supplementary Figures 3 and 4 illustrate that perturbing one part of the action potential model has complex effects on the entire dynamics of the system.

In our spatial simulations of the PV sleeve we use the simplified Kirchhoff network model (SKNM)^42^. This is a cell-based model as apposed to the homogenized bidomain and monodomain models often used to model cardiac tissue^43–47^. As such, the model represents the individual cardiomyocytes of the tissue and is able to directly represent properties varying between cardiomyocytes (e.g., whether they are PV or LA cardiomyocytes) as well as heterogeneities in the gap junction coupling between individual cells. Both of these properties are hard to straightforwardly represent using the bidomain or monodomain models^48^. On the other hand, the SKNM is a simplification of the somewhat more accurate Kirchhoff’s network model (KNM)^49^, and considerably less detailed than the extracellular-membrane-intracellular (EMI) model^50–52^ previously used to represent the PV sleeve in a cell-based manner^33^.

Compared to KNM, the SKNM represents the extracellular potential in a more simplified manner, but the difference between KNM and SKNM has been found to be relatively small in relevant test cases (see refs. ^42,53^ and Supplementary Note 3). Furthermore, KNM/SKNM have been found to be good approximations of the EMI model in some relevant test cases of atrial^49^ and ventricular tissue^53^. Nevertheless, the EMI model is a more accurate and detailed model than KNM and SKNM. For instance, the EMI model offers sub-cellular resolution, whereas KNM and SKNM has a resolution on the level of a single cell. Therefore, of these three models, EMI is the only model able to represent a non-uniform distribution of ion channels on the cell membrane, which has been observed in cardiomyocytes^54–56^ and found to affect properties of conduction^53,57,58^. Furthermore, only the EMI model is able to represent potential ephaptic coupling between neighbouring cardiomyocytes^57,59^.

In terms of computational efforts, the simulation time for SKNM is considerably longer for KNM than for SKNM. A 500 ms simulation of the PV sleeve requires 5 min of simulation time for SKNM and 6 h for KNM. For the EMI model, the same simulation takes 8 days^33^. Consequently, it is possible to perform much larger numbers of simulations using SKNM than EMI, and we have therefore chosen to use SKNM in our computations.

The proposed model aligns well with established characteristics of atrial tissue during AF, such as increased intracellular calcium concentration^16^, reduced calcium current^11,17–19^, shortened APD^20^, and the presence of stable re-entry waves^60^. Nonetheless, the model does not encompass several aspects of AF development. *First*, the initiation phase in our model (see Fig. 1) is simplified by forced electrical stimulation, intended to simulate rapid ectopic beats. Such initiations are recognized^13,15^ and can be modeled mathematically^61–63^. Our simplified approach do not fully capture the complexity of the actual physiological events. *Second*, our spatial simulations involve simplifications both in terms of considering a PV sleeve isolated from surrounding tissue and in terms of applying a specified stimulation current combined with a temporary directional block (see Fig. 2b) to enable the initiation of a one-directional traveling wave. The spatial simulations should therefore mainly be seen as investigations of the *potential for* re-entry around the PV sleeve under different conditions. *Third*, the applied model predicts an unrealistic stability of the re-entry waves; in reality, most arrhythmias in living tissue self-terminate, potentially due to stochastic perturbations not currently represented in the model. *Fourth*, the remodeling of other ionic currents is not addressed as mentioned above. *Fifth*, the model assumes constant functional changes as in Fig. 8; however, these changes can dynamically occur in vivo, suggesting that the assumption of constancy oversimplifies the physiological reality.

In conclusion, we have presented a computational model that demonstrates a possible path from paroxysmal AF episodes to sustained AF. The path appears to be robust in the sense that re-entry is obtained under a series of reasonable perturbations to the parameters. The model predicts that the occurrence of re-entry is proportional to the frequency and duration of the rapid firing. The occurrence also increases when the gap junction coupling is decreased and when the diameter of the pulmonary vein is increased. Finally, it increases as the fraction of pulmonary vein myocytes in relation to left atrial myocytes increases. The progression of atrial fibrillation, as depicted by the vicious cycle in Fig. 1, underscores the critical importance of early intervention in this cycle. The likelihood of successful repair diminishes as the disease advances, highlighting the necessity of timely therapeutic measures.

## Methods

In this section, we describe the models and methods used in the study. We describe the model used to represent the PV sleeve and the membrane models used to model the membrane dynamics of LA and PV cardiomyocytes. In addition, we describe the model used for the regulation of the number of *I*_CaL_ channels. Finally, we present the numerical methods used in the simulations.

### Cell-based mathematical models

In order to investigate the susceptibility of developing a re-entrant wave around the PV sleeve, we apply a cell-based mathematical model of the PV sleeve. We use the same set-up as that used to investigate the effect of mutations associated with AF in ref. ^33^. However, we will apply the computationally efficient ’simplified Kirchhoff’s network model’ (SKNM)^42^ as opposed to the more detailed and considerably more computationally expensive extracellular-membrane-intracellular (EMI) model (see, e.g.,^50,52,57,64–66^) applied in ref. ^33^. The SKNM is a simplification of Kirchhoff’s network model (KNM), and KNM has been shown to provide good approximations of the EMI model in simulations of cardiac tissue^49^.

The SKNM is derived from KNM based on the same simplifying assumption as that used to derive the monodomain model from the bidomain model of cardiac electrophysiology, and it has been shown that SKNM in several cases is a good approximation of KNM, see ref. ^42^. In Supplementary Note 3, we compare the solution of KNM and SKNM for some simulations of the PV sleeve, and we confirm that the solutions of the two models are similar also in this case (see Supplementary Fig. 5).

As mentioned above, the change of model from EMI to SKNM results in a considerable reduction in the required computational efforts. In ref. ^33^, a 500 ms EMI model simulation of the PV sleeve required a simulation time of about 8 days. Using SKNM, we are in this study able to perform the same simulation in about 5 min using the same computer. For comparison, the corresponding KNM simulations required a simulation time of about 6 hours. The reduced CPU efforts are important since they allow for large numbers of simulations, which will be necessary in order to investigate under what conditions sustainable re-entry will appear.

#### SKNM equations

The SKNM equations are given by

1 $${C}_{m}\frac{d{v}^{k}}{dt}=\frac{\lambda }{{A}_{m}(1+\lambda )}\mathop{\sum}\limits_{j\in {N}_{k}}{G}_{i}^{j,k}({v}^{j}-{v}^{k})-{I}_{{{{\rm{ion}}}}}^{k}({v}^{k},{s}^{k}),$$

2 $$\frac{d{s}^{k}}{dt}={F}_{k}({s}^{k},{v}^{k}).$$

Here, *C*_*m*_ is the specific membrane capacitance (in *μ*F/cm^2^), *A*_*m*_ is the membrane area of one cell (in cm^2^), *v*_*k*_ is the membrane potential of cell *k* (in mV), *N*_*k*_ is the collection of all the neighbors of cell *k*, ${G}_{j}^{j,k}$ is the intracellular conductance between cells *k* and *j* (in mS), and *λ* is a scaling factor involved in the derivation of SKNM from KNM (see ref. ^42^). This parameter will be defined below. Moreover, ${I}_{{{{\rm{ion}}}}}^{k}$ is the current density though ion channels, pumps and exchangers in the membrane of cell *k* (in *μ*A/cm^2^), *s*^*k*^ is a set of additional state variables involved in the computation of ${I}_{{{{\rm{ion}}}}}^{k}$, including gating variables for the ion channels and ionic concentrations, and *F*_*k*_ govern the dynamics of these variables. The models used for ${I}_{{{{\rm{ion}}}}}^{k}$, *s*^*k*^ and *F*_*k*_ are described below, and the details are provided in Supplementary Note 4.

#### SKNM parameters

The default parameters used in the SKNM simulations of this study based on ref. ^33^ are given in Table 1. Here, *l*_*l*_ is the cell length in the longitudinal direction and *l*_*t*_ is the maximal cell width in the transverse direction. Similarly, ${R}_{{{{\rm{gap}}}}}^{* ,l}$ and ${R}_{{{{\rm{gap}}}}}^{* ,t}$ are the default gap junction resistances in the longitudinal and transverse directions, respectively.

**Table 1:** Default parameter values used in the SKNM simulations

Parameter	Value
*C*_*m*_	1 *μ*F/cm^2^
*A*_*m*_	5 ⋅ 10^−5^ cm^2^
*σ*_*i*_	4 mS/cm
*σ*_*e*_	20 mS/cm
*δ*_*e*_	0.2
*l*_*l*_	120 μm
*l*_*t*_	14 μm
*λ*	3.25
${R}_{{{{\rm{gap}}}}}^{* ,l}$	380 kΩ
${R}_{{{{\rm{gap}}}}}^{* ,t}$	760 kΩ

The SKNM parameters ${G}_{i}^{j,k}$ and *λ* are defined as described in ref. ^42^. In short,

3 $${G}_{i}^{j,k}=\frac{1}{\frac{{l}_{j,k}}{{\delta }_{i}{A}_{j,k}{\sigma }_{i}}+\frac{1}{{G}_{g}^{j,k}}},$$

4 $${G}_{e}^{j,k}={\delta }_{e}\frac{{A}_{j,k}{\sigma }_{e}}{{l}_{j,k}},$$

5 $$\lambda =\frac{{\sum }_{j,k}{G}_{e}^{j,k}{G}_{i}^{j,k}\frac{{l}_{j,k}^{2}}{{A}_{j,k}^{2}}}{{\sum }_{j,k}{\left({G}_{i}^{j,k}\right)}^{2}\frac{{l}_{j,k}^{2}}{{A}_{j,k}^{2}}}.$$

Here, *l*_*j*,*k*_ is the distance between the centers of cells *k* and *j* (in cm), *δ*_*e*_ (unitless) is the average extracellular volume fraction, *δ*_*i*_ = 1 − *δ*_*e*_ (unitless) is the average intracellular volume fraction, *A*_*j*,*k*_ (in cm^2^) is the average cross-sectional areas of compartments *j* and *k* (containing both intracellular and extracellular space), *σ*_*i*_ and *σ*_*e*_ are the intracellular and extracellular conductivities, respectively, (in mS/cm), ${G}_{g}^{j,k}=1/{R}_{g}^{j,k}$ is the conductance of the gap junctions connecting cells *j* and *k* (in mS), and ${R}_{g}^{j,k}$ is the corresponding gap junction resistance (in kΩ).

To represent the slow and complex conduction observed in the PV sleeve^67^, we vary the value of the gap junction resistance randomly for each cell connection like in ref. ^33^. More specifically, for each cell connection, we draw a random number, *β*^*j*,*k*^, between 0 and 1 and let the gap junction resistance be given by

6 $${R}_{g}^{j,k}={\beta }^{j,k}\cdot {R}_{g}^{* ,x}+(1-{\beta }^{j,k})\cdot 30{R}_{g}^{* ,x},$$

where ${R}_{g}^{* ,x}$ is either ${R}_{g}^{* ,l}$ or ${R}_{g}^{* ,t}$ specified in Table 1, depending on whether the connection between cells *j* and *k* is in the longitudinal or transverse cell directions.

#### The mesh defined by cardiomyocytes

**Fig. 10:**
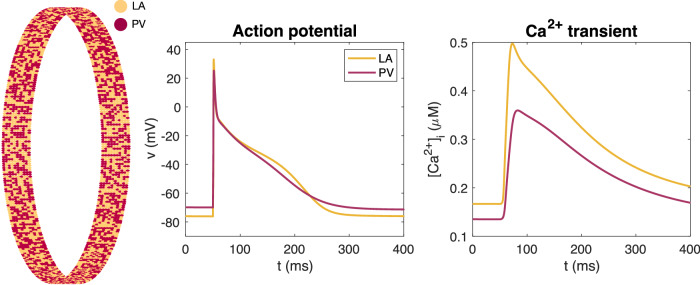
Mix of PV and LA cardiomyocytes. Illustration of the mix of LA and PV cardiomyocytes in the spatial representation of the PV sleeve. The left panel shows an overview of the location of LA and PV myocytes in the PV sleeve. The density of certain ion channels are different for the PV and LA myocytes, resulting in the displayed differences in the action potential and calcium transients in single-cell simulations of the two cell types, shown in the center and right panels. The full model formulation is found in Supplementary Note 4.

In SKNM, the location of the cells define the computational mesh. In our simulations, we consider a collection of cells located in a cylinder at the outlet of a pulmonary vein. We use the same PV sleeve geometry as in ref. ^33^. That is, each cell is assumed to have a maximal diameter of 14 μm and a length of 120 μm, and the cells are connected in the longitudinal direction around the cylinder. In addition, 10 rows of connected cells are located along the longitudinal axis of the cylinder. In the default case, the PV sleeve is assumed to have a diameter of 1.5 cm, similar to diameters measured for human pulmonary veins^68^. This diameter corresponds to 393 cell lengths. Thus, in the default case, the domain consists of 393 × 10 cells, see leftmost panel of Fig. 10.

### The PV-LA cell-based model

In the SKNM collection of cells, we assume that the cells are either ordinary left atrial (LA) cardiomyocytes, or pulmonary vein (PV) cardiomyocytes. These two cell types have been shown to have some differences in the density of some types of ion channels^69^. The differences are incorporated into the model used to define the membrane dynamics, ${I}_{{{{\rm{ion}}}}}^{k}$ and *F*_*k*_. We use the membrane models from ref. ^33^ with a few parameter adjustments to represent the two cell types. These adjustments will be described below. In addition, the details of the membrane models are provided in Supplementary Note 4. In Fig. 10 we show the action potential and calcium transients of the two versions of the membrane model, as well as the spatial distribution of cell types in the PV sleeve cylinder in the default case. In the default case, we consider 50% PV cells and 50% LA cells.

### Modeling the expression of calcium channels

A model of the expression of ion channels carrying the *I*_CaL_ current can be written on the form

7 $${\tau }_{m}\frac{dm}{dt}={c}^{* }-c,$$

8 $${\tau }_{n}\frac{dn}{dt}=m-n,$$

see refs. ^24,28,32^. In this model, *τ*_*m*_ (in mMms) and *τ*_*n*_ (in ms) are time constants, *c* (in mM) is the cytosolic calcium concentration, and c^*^ (in mM) is the target level of the cytosolic calcium concentration. Furthermore, *m* = *m*(*t*) and *n* = *n*(*t*) denote the relative changes of the number of the messenger RNAs, *M* = *M*(*t*), and the number of expressed calcium ion channel proteins in the cell membrane, *N* = *N*(*t*), respectively. Specifically, we let *M*_0_ and *N*_0_ denote the default values of *M* and *N*, and therefore *M*(*t*) = *m*(*t*)*M*_0_ and *N*(*t*) = *n*(*t*)*N*_0_. The number of ion channels carrying the *I*_CaL_-current affects the strength of the current as follows,

9 $${I}_{{{{\rm{CaL}}}}}=\frac{n{N}_{0}}{A{C}_{m}}\cdot o\cdot {i}_{{{{\rm{CaL}}}}}.$$

Here, *C*_*m*_ is the specific membrane capacitance (in *μ*F/cm^2^), *A* is the area of the cell membrane (in cm^2^), *o* is the (unitless) open probability, and *i*_CaL_ is the average current through a single open calcium channel (in *μ*A).

One disadvantage of this model is that it does not impose a natural upper or lower bound on the number of ion channels. In ref. ^28^ it was shown that the model (7)–(8) can be accurately approximated by the following scalar model which includes bounds on the number of ion channels,

10 $${\tau }_{n}\frac{dn}{dt}=({c}^{* }-c)H(c,n),$$

11 $$H(c,n)=h(n,{n}_{-},{\varepsilon }_{n})h(c,{c}^{* },{\varepsilon }_{c})+h({n}_{+},n,{\varepsilon }_{n})h({c}^{* },c,{\varepsilon }_{c}),$$

12 $$h(a,b,\varepsilon )=\frac{1}{2}\left(1+\tanh \left(\frac{a-b}{\varepsilon }\right)\right).$$

Here, *n* = *n*_−_ and *n* = *n*_+_ defines lower and upper bounds for the relative changes in the number of ion channels *N* = *N*(*t*). A plot of the function *H* are given in Supplementary Note 4 In our computations, we use the model (10)–(12) to model the regulation of the number of *I*_CaL_ channels.

### Parameterization of the model for channel expression

In the computations presented here, we have set the lower bound of channels to *n*_−_ = 0.28, motivated by the fact that *I*_CaL_ has been observed to be reduced to about 28% in patients with persistent AF^70^. In order to achieve a value of *n* close to this experimental value in the LA model following 600 beats per minute (bpm) pacing, we also needed to make some adjustments of the membrane model parameterizations. Specifically, ${\bar{I}}_{{{{\rm{NaCa}}}}}$ is reduced by 40%, *g*_Kr_ is reduced by 20% and the calcium buffer concentrations ${B}_{{{{\rm{tot}}}}}^{c}$ and ${B}_{{{{\rm{tot}}}}}^{sl}$ are both increased by 40% in the updated versions of the models.

For the remaining channel expression model parameterization, we have re-used the parameter *n*_+_ = 3 from ref. ^28^, but since *n* is decreasing and not increasing in our simulations, this value does not influence the results of this study. Furthermore, the value of *τ*_*n*_ is set to 30,000 mMms. This value was chosen because it resulted in a reduction of *I*_CaL_ of about 50% after approximately 12 h of rapid pacing (600 bpm) in the left atrial (LA) membrane model, consistent with experimental measurements of *I*_CaL_ in rabbit right atria at 600 bpm pacing from ref. ^17^. The value of c^*^ for LA and PV is defined as the average value of the cytosolic calcium concentration over one second in the default versions of the LA and PV membrane models paced at 1 beat per second (1 bps).

### Numerical implementations

We perform simulations using SKNM (1)–(2) coupled to the membrane models as described above as well as simulations of the single-cell membrane models in the form

13 $${C}_{m}\frac{d{v}^{k}}{dt}=-{I}_{{{{\rm{ion}}}}}^{k}({v}^{k},{s}^{k}),$$

14 $$\frac{d{s}^{k}}{dt}={F}_{k}({s}^{k},{v}^{k}).$$

The single-cell membrane model simulations are performed in MATLAB using the ode15s solver. The SKNM simulations are done in C++ using a standard operator splitting of the linear and non-linear systems (see, e.g.,^71,72^) and a time step of Δ*t* = 0.001 ms. The linear part of the system is solved implicitly using the BiCGSTAB iterative solver from the MFEM library^73,74^, and the non-linear part is solved using the first-order Rush-Larsen method^75,76^ with code generated by the Gotran code generator^77^. We apply OpenMP parallelization^78^ for the solution of the non-linear equations. The computations are run on a Dell Precision 3640 Tower with an Intel Core processor (i9-10900 K, 3.7 GHz/5.4 GHz) with 10 cores, each with 2 threads.

### Computation of biomarkers

#### Computation of APD80

The action potential duration at 80% repolarization (APD80) used in Fig. 9 is computed by taking the average of the APD80 value computed for each cell in the tissue. For a single cell, the APD80 value is computed as the duration of time from the point in time when the membrane potential increases most rapidly during the upstroke of the action potential until the time when the membrane potential is 80% repolarized.

#### Computation of CV

The conduction velocity is computed by dividing the distance that the excitation wave must travel between two cells located in the center of the PV sleeve divided by the duration of time between the first cell and the second cell reaches a membrane potential above −20 mV. The distance that the excitation wave must travel between the two cells is 2.4 cm.

#### Computation of L

The total length that the excitation wave may travel around the PV sleeve, L, is computed by *N*_*l*_ × *l*_*l*_, where *N*_*l*_ is the number of cells located around the PV sleeve and *l*_*l*_ is the length of one cell.

### Disclosure of writing assistance

During the preparation of this manuscript, the authors utilized the ChatGPT4 language model to enhance the language quality for contributions from non-native English speakers. Subsequent to this automated assistance, the authors rigorously reviewed and edited the manuscript to ensure its accuracy and integrity. The authors assume full responsibility for the content of the publication.

### Supplementary information

Supplementary Information

## Data Availability

The data generated in this study are publicly available at Zenodo: 10.5281/zenodo.11241241^79^.

## Code availability

The code generated in this study are publicly available at Zenodo: 10.5281/zenodo.11241241^79^.
