# Supplementary material for: A possible path to persistent re-entry waves at the outlet of the left pulmonary vein
Source: NPJ Syst Biol Appl. 2024 Jul 23;10:79. doi: 10.1038/s41540-024-00406-9 (PMC11266599; doi:10.1038/s41540-024-00406-9)
Supplement: Supplementary file 1 — Supplementary Information [file 41540_2024_406_MOESM1_ESM.pdf]

Supplementary Information for

A possible path to persistent re-entry waves at the outlet of the left pulmonary vein

Supplementary Note 1: Properties of the LA and PV membrane models with a fixed number of channels

In this supplementary note, we investigate some properties of the left atrial (LA) and pulmonary vein (PV) cardiomyocyte membrane models described in Supplementary Note 5. We consider the version of the models without protein regulation (i.e., with the number of calcium channels fixed at $n = 1$).

Effect of rapid pacing on the cytosolic calcium concentration

In Supplementary Figure 1, we investigate the effect of the pacing frequency on the average cytosolic calcium concentration between each stimulation. We consider pacing frequencies ranging from the default 1 beats per second (bps) up to 10 bps. We observe that after a few minutes of rapid pacing, the cytosolic calcium concentration increases and that the increase in calcium concentration is larger for a higher pacing frequency.

Effect of adjusting currents on the cytosolic calcium concentration

In Supplementary Figure 2, we report the percent change in the average cytosolic calcium concentration resulting from a 20% increase in each type of ion channel, pump and exchanger in the cell membrane for the LA and PV membrane models without protein regulation. We observe that increasing

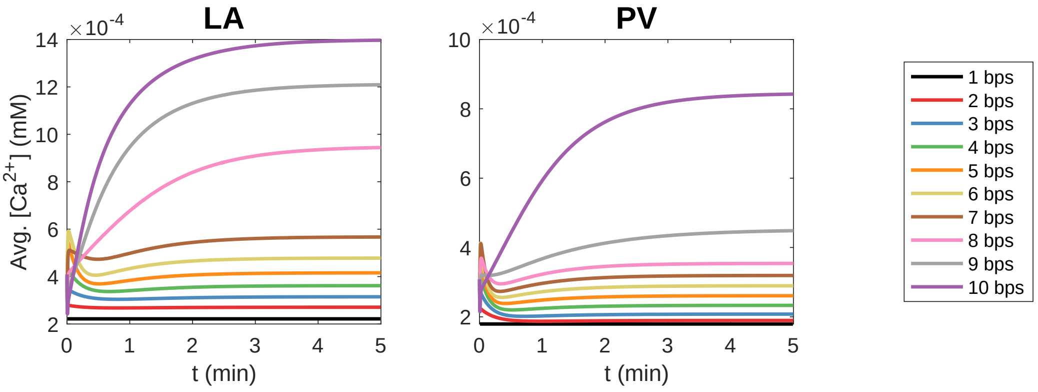

Supplementary Figure 1: Effect of rapid pacing on the intracellular calcium concentration in the PV and LA membrane models without protein regulation. The figure shows the time evolution of the average cytosolic calcium concentration as a function of time for pacing frequencies ranging from 1 beat per second (bps) to 10 bps.

the number of channels responsible for the fast sodium current (I_{Na}), the late sodium current (I_{NaL}) or the background sodium current slightly increases the intracellular calcium concentration. Furthermore, increasing the channels responsible for the L-type calcium current (I_{CaL}) or the background calcium current (I_{bCa}) results in a considerable increase in the intracellular calcium concentration. On the other hand, increasing the number calcium pumps (I_{pCa}), sodium-calcium exchangers (I_{NaCa}), sodium-potassium pumps (I_{NaK}), background chloride channels (I_{bCl}), or I_{Kr}, I_{Ks}, I_{to}, or I_{Kur} potassium channels, causes an decrease in the cytosolic calcium concentration. The effect of increasing the number of I_{K1} and I_{f} channels on the cytosolic calcium concentration is small, and differs between the PV and LA versions of the model.

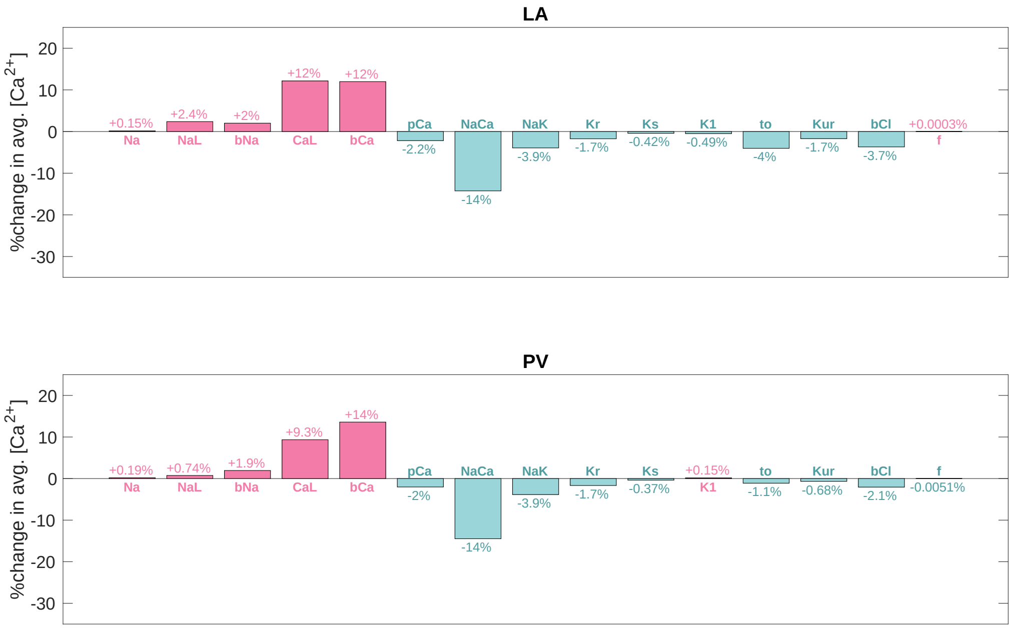

Supplementary Figure 2: Effect on the intracellular calcium concentration of increasing the number of different types of membrane proteins. For the LA and PV membrane models without protein regulation, we report the percent change in the average cytosolic calcium concentration resulting from a 20% increase in each type of ion channel, pump and exchanger in the cell membrane. The concentrations are recorded 1 h after the parameter change was applied.

Supplementary Note 2: Effect of rapid pacing on all currents

In Supplementary Figure 3, we show the baseline LA membrane currents during an action potential in addition to the membrane currents following 1 hour and 72 hours of 10 beats per second rapid pacing. In these simulations, the number of L-type calcium channels are regulated by the cytosolic calcium concentration (see Figure 4 in the paper). In Supplementary Figure 4 we show the same results for the PV case.

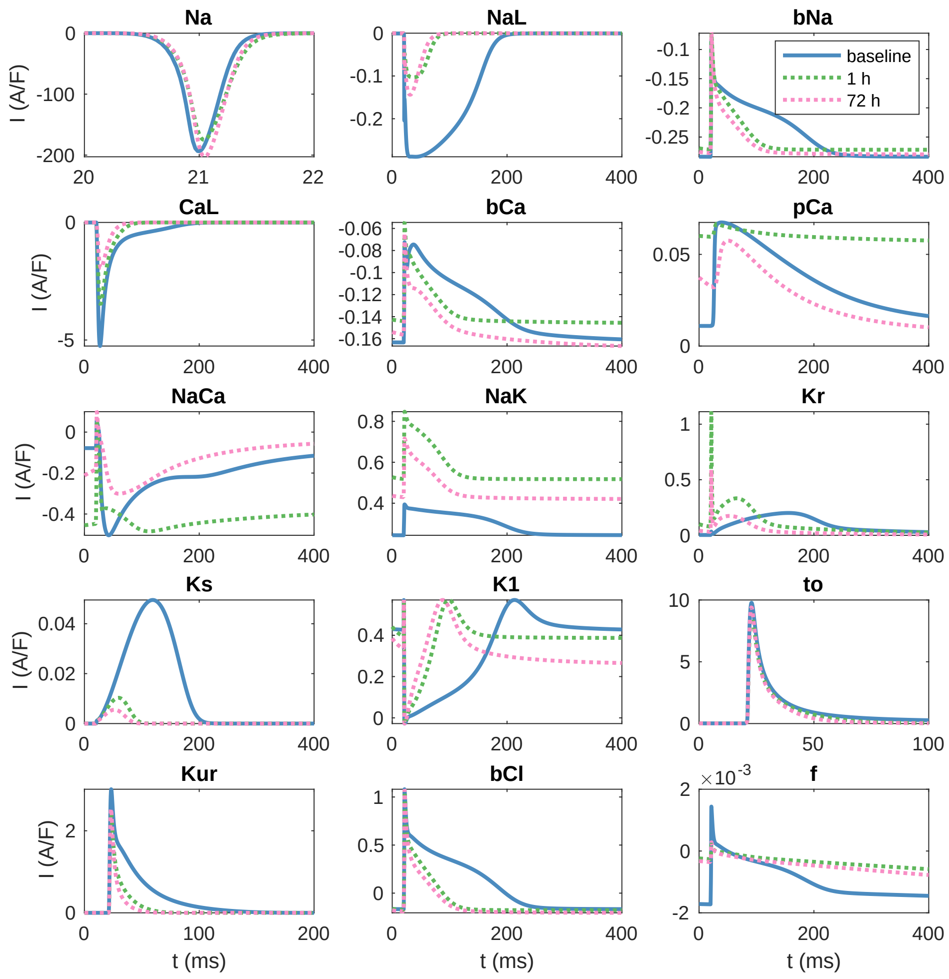

Supplementary Figure 3: Effect of rapid pacing and L-type calcium channel regulation on all the membrane currents during an LA action potential. We consider 10 bps pacing and record the currents after 1 hour and 72 hours.

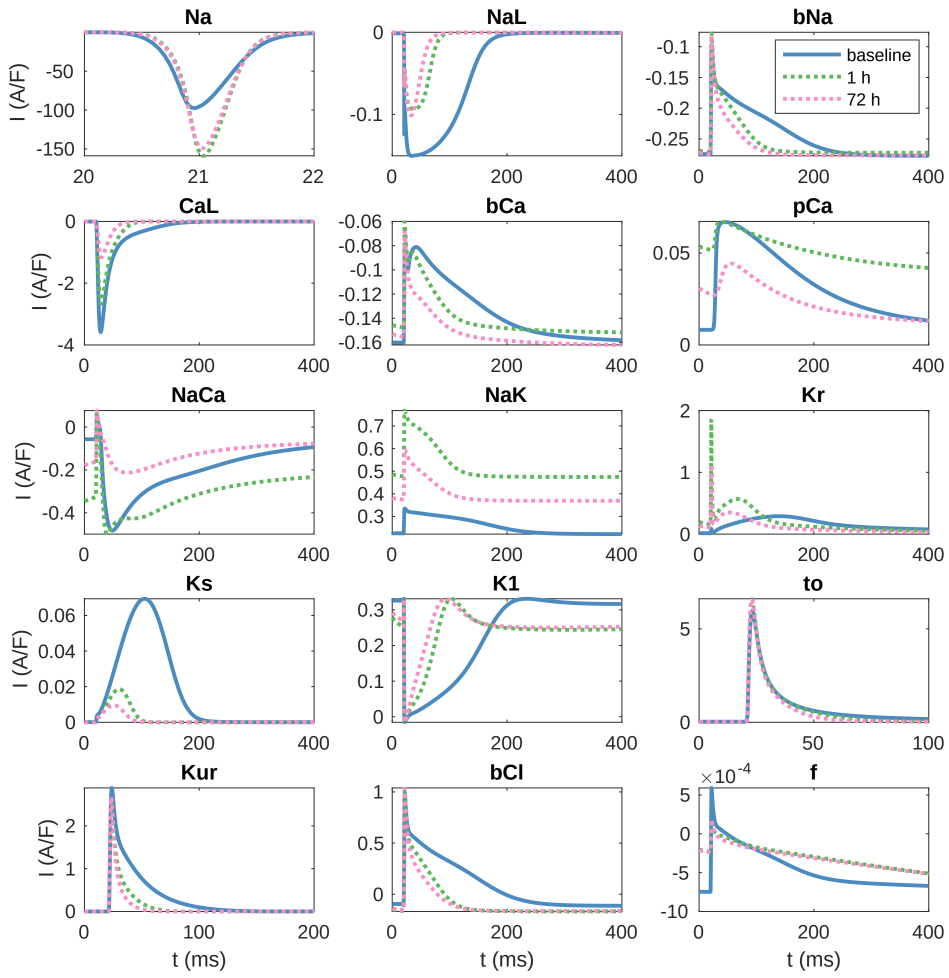

Supplementary Figure 4: Effect of rapid pacing and L-type calcium channel regulation on all the membrane currents during a PV action potential. We consider 10 bps pacing and record the currents after 1 hour and 72 hours.

Supplementary Note 3: Comparison of KNM and SKNM solutions

Supplementary Figure 5 shows a comparison of the solutions of the Kirchhoff network model (KNM) and the simplified Kirchhoff network model (SKNM) [1, 2].

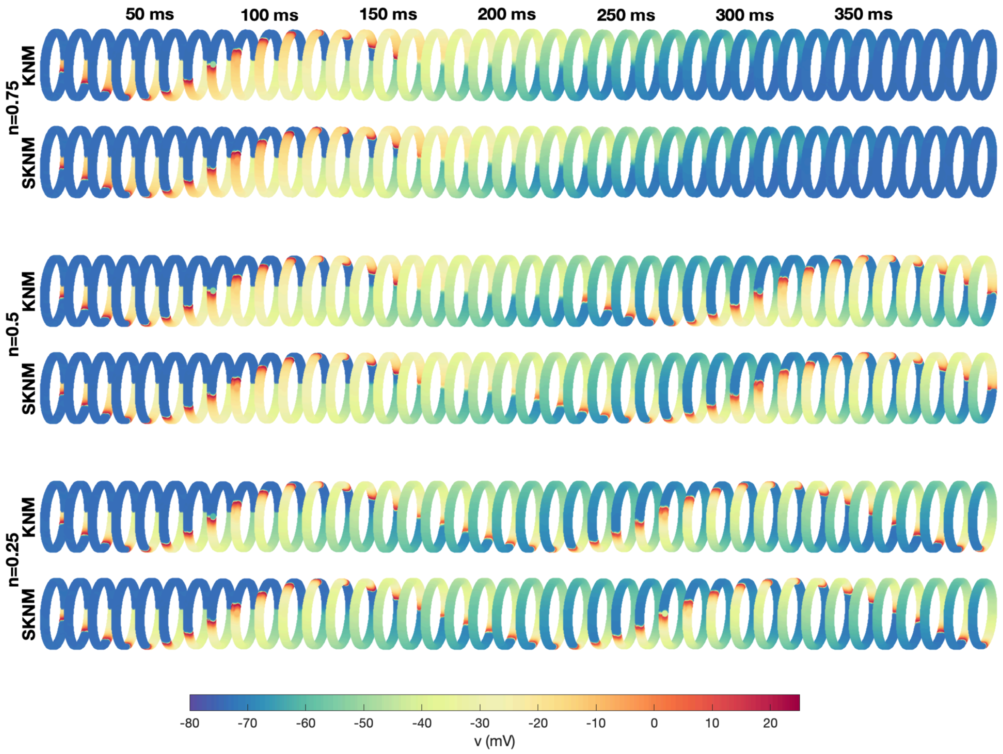

Supplementary Figure 5: Comparison of KNM and SKNM solutions. The figure shows the membrane potential for the cells in the PV sleeve cylinder at some different points in time after a stimulation is applied for some KNM and SKNM simulations of $n = 0.75$, $n = 0.5$ and $n = 0.25$. We observe that the two models produce quite similar solutions, but that the conduction velocity does not seem to be entirely identical for the two models.

Supplementary Note 4: Membrane model formulation

In this supplementary note, we describe the formulation of the human left atrial (LA) and pulmonary vein (PV) cardiomyocyte versions of our membrane model. Here, the membrane potential (v) is given in units of mV, and the Ca^{2+} and Na^{+} concentrations are given in units of mM. All currents are given in units of A/F, and the ionic fluxes are expressed as mmol/ms per total cell volume (i.e., in units of mM/ms). Time is given in ms. The parameters of the model are all given in Supplementary Tables 1–7. In particular, the adjustment factors used to scale the model from the left atrial version to the pulmonary vein version of the model are found in Supplementary Table 4.

Note that the model formulation is very similar to the base model from [3]. The only difference is that $\bar{I}_{\text{NaCa}}$ is decreased by 40%, g_{Kr} is decreased by 20% and the calcium buffer concentrations B^{c}_{tot} and B^{sl}_{tot} are increased by 40% compared to the version in [3]. In addition, a model for the number of I_{CaL} channels on the cell membrane has been added.

Membrane potential

In the membrane model formulation, the membrane potential is governed by

$$\begin{aligned}\frac{dv}{dt} = -(&I_{\text{Na}} + I_{\text{NaL}} + I_{\text{CaL}} + I_{\text{to}} + I_{\text{Kr}} + I_{\text{Ks}} + I_{\text{K1}} + I_{\text{NaCa}} + I_{\text{NaK}} \\ &+ I_{\text{pCa}} + I_{\text{bCa}} + I_{\text{bCl}} + I_{\text{bNa}} + I_{\text{f}} + I_{\text{Kur}} + I_{\text{stim}}),\end{aligned} \tag{1}$$

where I_{Na}, I_{NaL}, I_{CaL}, I_{to}, I_{Kr}, I_{Ks}, I_{K1}, I_{NaCa}, I_{NaK}, I_{pCa}, I_{bCl}, I_{bCa}, I_{bNa}, I_{f}, and I_{Kur} are transmembrane currents that will be specified below and I_{stim} is an applied stimulus current. Unless otherwise specified, we let I_{stim} be given as a constant current of size -40 A/F applied for 1 ms.

Membrane currents

In general, the currents through the voltage-gated ion channels within the myocyte membrane are given on the form

$$I = go(v - E),$$

where g is the channel conductance, v is the membrane potential and E is the equilibrium potential of the channel. Moreover, o is the open probability of the channels, which is given on the form $o = \prod_i z_i$, where z_i are gating

variables. These gating variables are either given as an explicit function of the membrane potential or governed by equations of the form

$$z_i' = \frac{1}{\tau_{z_i}}(z_{i,\infty} - z_i). \tag{2}$$

The parameters τ_{z_i} and $z_{i,\infty}$ are specified for each of the gating variables of the model in Supplementary Table 10.

Fast sodium current (I_{Na}) The formulation of the fast sodium current is based on the model formulation given in [4] and is given by

$$I_{\mathrm{Na}} = g_{\mathrm{Na}} o_{\mathrm{Na}} (v - E_{\mathrm{Na}}), \tag{3}$$

where the open probability is given by

$$o_{\mathrm{Na}} = m^3 j, \tag{4}$$

and m and j are gating variables governed by equations of the form (2).

Late sodium current (I_{NaL}) The formulation of the late sodium current, I_{NaL}, is based on [5] and is given by

$$I_{\mathrm{NaL}} = g_{\mathrm{NaL}} o_{\mathrm{NaL}} (v - E_{\mathrm{Na}}), \tag{5}$$

where the open probability is given by

$$o_{\mathrm{NaL}} = m_L h_L, \tag{6}$$

and m_L and h_L are gating variables governed by equations of the form (2).

Transient outward potassium current (I_{to}) The formulation of the transient outward potassium current, I_{to}, is based on [6] and is given by

$$I_{\mathrm{to}} = g_{\mathrm{to}} o_{\mathrm{to}} (v - E_{\mathrm{K}}), \tag{7}$$

where the open probability is given by

$$o_{\mathrm{to}} = q_{\mathrm{to}} r_{\mathrm{to}}, \tag{8}$$

and q_{to} and r_{to} are gating variables governed by equations of the form (2).

Rapidly activating potassium current (I_{Kr}) The rapidly activating potassium current, I_{Kr}, is formulated as a Markov model, based on [7]. The formulation has been fitted to data of WT and N588K I_{Kr} currents from [8]. The current is given by

$$I_{\mathrm{Kr}} = g_{\mathrm{Kr}} \sqrt{\frac{[\mathrm{K}^+]_e}{[\mathrm{K}^+]_e^b}} \frac{T - 275 \text{ K}}{35 \text{ K}} o_{\mathrm{Kr}} (v - E_K), \tag{9}$$

where o_{Kr} is modelled by a Markov model of the form

$$c_{\mathrm{Kr},1} \underset{\beta_{\mathrm{Kr},1}}{\overset{\alpha_{\mathrm{Kr},1}}{\rightleftharpoons}} c_{\mathrm{Kr},2} \underset{\beta_{\mathrm{Kr},2}}{\overset{\alpha_{\mathrm{Kr},2}}{\rightleftharpoons}} c_{\mathrm{Kr},3} \underset{\beta_{\mathrm{Kr},3}}{\overset{\alpha_{\mathrm{Kr},3}}{\rightleftharpoons}} o_{\mathrm{Kr}} \underset{\beta_{\mathrm{Kr},4}}{\overset{\alpha_{\mathrm{Kr},4}}{\rightleftharpoons}} i_{\mathrm{Kr}}. \tag{10}$$

Here, the dynamics of the closed states $c_{\mathrm{Kr},1}$, $c_{\mathrm{Kr},2}$, and $c_{\mathrm{Kr},3}$, the open state o_{Kr}, and the inactivated state i_{Kr} are given by

$$\frac{c_{\mathrm{Kr},1}}{dt} = \beta_{\mathrm{Kr},1} c_{\mathrm{Kr},2} - \alpha_{\mathrm{Kr},1} c_{\mathrm{Kr},1}, \tag{11}$$

$$\frac{c_{\mathrm{Kr},2}}{dt} = \alpha_{\mathrm{Kr},1} c_{\mathrm{Kr},1} + \beta_{\mathrm{Kr},2} c_{\mathrm{Kr},3} - (\alpha_{\mathrm{Kr},2} + \beta_{\mathrm{Kr},1}) c_{\mathrm{Kr},2}, \tag{12}$$

$$\frac{c_{\mathrm{Kr},3}}{dt} = \alpha_{\mathrm{Kr},2} c_{\mathrm{Kr},2} + \beta_{\mathrm{Kr},3} o_{\mathrm{Kr}} - (\alpha_{\mathrm{Kr},3} + \beta_{\mathrm{Kr},2}) c_{\mathrm{Kr},3}, \tag{13}$$

$$\frac{o_{\mathrm{Kr}}}{dt} = \alpha_{\mathrm{Kr},3} c_{\mathrm{Kr},3} + \beta_{\mathrm{Kr},4} i_{\mathrm{Kr}} - (\alpha_{\mathrm{Kr},4} + \beta_{\mathrm{Kr},3}) o_{\mathrm{Kr}}, \tag{14}$$

$$\frac{i_{\mathrm{Kr}}}{dt} = \alpha_{\mathrm{Kr},4} o_{\mathrm{Kr}} - \beta_{\mathrm{Kr},4} i_{\mathrm{Kr}}, \tag{15}$$

where the transition rates are given by provided in Supplementary Table 8.

Slowly activating potassium current (I_{Ks}) The formulation of the slowly activating potassium current, I_{Ks}, is based on [4] and is given by

$$I_{\mathrm{Ks}} = g_{\mathrm{Ks}} o_{\mathrm{Ks}} (v - E_{\mathrm{Ks}}), \tag{16}$$

where

$$o_{\mathrm{Ks}} = x_{\mathrm{Ks}}^2, \tag{17}$$

and the dynamics of x_{Ks} is governed by an equation of the form (2).

Inward rectifier potassium current (I_{K1}) The formulation of the inward rectifier potassium current, I_{K1}, is based on [9, 10], and is given by

$$I_{\mathrm{K1}} = g_{\mathrm{K1}} \sqrt{\frac{[\mathrm{K}^+]_\mathrm{e}}{[\mathrm{K}^+]_\mathrm{e}^b}} \left(\frac{0.059764(v - E_\mathrm{K} + 0.582854)}{0.754829 + e^{0.0767156(v - E_\mathrm{K} + 0.582854)}} - 0.0114237 \right).$$

Ultrarapid delayed rectifier potassium current (I_{Kur}) The formulation of the ultrarapid delayed rectifier potassium current, I_{Kur}, is based on [11, 12] and is given by

$$I_{\text{Kur}} = g_{\text{Kur}} f_{\text{Kur}} o_{\text{Kur}} (v - E_{\text{K}}), \tag{18}$$

where f_{Kur} is given by

$$f_{\text{Kur}} = 0.005 + \frac{0.05}{1 + e^{-\frac{v-15}{13}}} \tag{19}$$

and

$$o_{\text{Kur}} = x_{\text{Kur1}}^3 \cdot x_{\text{Kur2}}. \tag{20}$$

The dynamics of x_{Kur1} and x_{Kur2} are governed by equations of the form (2).

Hyperpolarization activated funny current (I_{f}) The formulation for the hyperpolarization activated funny current, I_{f}, is based on [6] and is given by

$$I_{\text{f}} = g_{\text{f}} o_{\text{f}} (v - E_{\text{f}}), \tag{21}$$

where

$$o_{\text{f}} = x_{\text{f}}, \tag{22}$$

and the dynamics of x_{f} is governed by an equation of the form (2).

L-type Ca^{2+} current (I_{CaL}) The formualtion for the L-type Ca^{2+} current, I_{CaL}, is based on the formulation in [4] and is given by

$$I_{\text{CaL}} = n g_{\text{CaL}} \left(Q_{10}^{\text{CaL}}\right)^{Q_p} o_{\text{CaL}} \frac{(2F)^2 v}{RT} \frac{0.341 c_d e^{\frac{2Fv}{RT}} - 0.341 c_e}{e^{\frac{2Fv}{RT}} - 1}, \tag{23}$$

where

$$o_{\text{CaL}} = df(1 - f_{\text{Ca}}), \tag{24}$$

and the dynamics of d, f and f_{Ca} are governed by equations of the form (2). Note that n is governed by (54) and represents the relative number of I_{CaL} channels.

Time-independent background currents (I_{bCa}, I_{bBa}, I_{bCl}) The formulation of the background currents, I_{bCa}, I_{bNa} and I_{bCl}, are based on [4] and are given by

$$I_{\text{bCa}} = g_{\text{bCa}} (v - E_{\text{Ca}}), \tag{25}$$

$$I_{\text{bNa}} = g_{\text{bNa}} (v - E_{\text{Na}}), \tag{26}$$

$$I_{\text{bCl}} = g_{\text{bCl}} (v - E_{\text{Cl}}). \tag{27}$$

Sodium-calcium exchanger current (I_{NaCa}) The formulation of the Na^+-Ca^{2+} exchanger current, I_{NaCa}, is based on [4] and is given by

$$I_{\mathrm{NaCa}} = \bar{I}_{\mathrm{NaCa}} \left(Q_{10}^{\mathrm{NaCa}}\right)^{Q_p} \frac{e^{\frac{\nu F v}{RT}}[\mathrm{Na}^+]_i^3 c_e - e^{\frac{(\nu-1)Fv}{RT}}[\mathrm{Na}^+]_e^3 c_{sl}}{s_{\mathrm{NaCa}}\left(1+\left(\frac{K_{\mathrm{act}}}{c_{sl}}\right)^2\right)\left(1+k_{\mathrm{sat}}e^{\frac{(\nu-1)Fv}{RT}}\right)}, \tag{28}$$

where

$$\begin{aligned} s_{\mathrm{NaCa}} = {} & K_{\mathrm{Ca},i}[\mathrm{Na}^+]_e^3 \left(1+\left(\frac{[\mathrm{Na}^+]_i}{K_{\mathrm{Na},i}}\right)^3\right) + K_{\mathrm{Na},e}^3 c_{sl}\left(1+\frac{c_{sl}}{K_{\mathrm{Ca},i}}\right) \\ & + K_{\mathrm{Ca},e}[\mathrm{Na}^+]_i^3 + [\mathrm{Na}^+]_i^3 c_e + [\mathrm{Na}^+]_e^3 c_{sl}. \end{aligned}$$

Sarcolemmal Ca^{2+} pump current (I_{pCa}) The formulation of the current through the sarcolemmal Ca^{2+} pump, I_{pCa}, is based on [4] and is given by

$$I_{\mathrm{pCa}} = \bar{I}_{\mathrm{pCa}} \left(Q_{10}^{\mathrm{pCa}}\right)^{Q_p} \frac{c_{sl}^2}{K_{\mathrm{pCa}}^2 + c_{sl}^2}. \tag{29}$$

Sodium-potassium pump current (I_{NaK}) The current through the Na^+-K^+ pump, I_{NaK}, is based on [4] and is given by

$$I_{\mathrm{NaK}} = \bar{I}_{\mathrm{NaK}} \left(Q_{10}^{\mathrm{NaK}}\right)^{Q_p} \frac{f_{\mathrm{NaK}}}{1+\left(\frac{K_{\mathrm{Na,i}}^{\mathrm{NaK}}}{[\mathrm{Na}^+]_{\mathrm{i}}}\right)^4} \frac{[\mathrm{K}^+]_{\mathrm{e}}}{[\mathrm{K}^+]_{\mathrm{e}} + K_{\mathrm{K,e}}}, \tag{30}$$

where

$$f_{\mathrm{NaK}} = \frac{1}{1+0.12e^{-0.1\frac{Fv}{RT}}} + \frac{0.037}{7}\left(e^{\frac{[\mathrm{Na}^+]_{\mathrm{e}}}{67}} - 1\right)e^{-\frac{Fv}{RT}}. \tag{31}$$

Intracellular [Ca^{2+}] dynamics

The Ca^{2+} dynamics are governed by

$$\frac{dc_d}{dt} = \frac{1}{V_d}(J_{\mathrm{CaL}} - J_d^b - J_d^c), \qquad \frac{db_d}{dt} = \frac{1}{V_d}J_d^b, \tag{32}$$

$$\frac{dc_{sl}}{dt} = \frac{1}{V_{sl}}(J_e^{sl} - J_{sl}^c - J_{sl}^b + J_s^{sl}), \qquad \frac{db_{sl}}{dt} = \frac{1}{V_{sl}}J_{sl}^b, \tag{33}$$

$$\frac{dc_c}{dt} = \frac{1}{V_c}(J_{sl}^c + J_d^c - J_c^n - J_c^b), \qquad \frac{db_c}{dt} = \frac{1}{V_c}J_c^b, \tag{34}$$

$$\frac{dc_s}{dt} = \frac{1}{V_s}(J_n^s - J_s^{sl} - J_s^b), \qquad \frac{db_s}{dt} = \frac{1}{V_s}J_s^b, \tag{35}$$

$$\frac{dc_n}{dt} = \frac{1}{V_n}(J_c^n - J_n^s). \tag{36}$$

Here, c_d is the concentration of free Ca^{2+} in the dyad, b_d is the concentration of Ca^{2+} bound to a buffer in the dyad, c_{sl} is the concentration of free Ca^{2+} in the sub-sarcolemmal (SL) compartment, b_{sl} is the concentration of Ca^{2+} bound to a buffer in the SL compartment, c_c is the concentration of free Ca^{2+} in the bulk cytosol, b_c is the concentration of Ca^{2+} bound to a buffer in the bulk cytosol, c_s is the concentration of free Ca^{2+} in the junctional sarcoplasmic reticulum (jSR), b_s is the concentration of Ca^{2+} bound to a buffer in the jSR, and c_n is the concentration of free Ca^{2+} in the network sarcoplasmic reticulum (nSR). The expressions for the fluxes are specified below.

Ca^{2+} fluxes

Flux through the SERCA pumps The flux from the bulk cytosol to the nSR through the SERCA pumps is based on [4] and given by

$$J_c^n = \bar{J}_{\mathrm{SERCA}} \left(Q_{10}^{\mathrm{SERCA}}\right)^{Q_p} \frac{\left(\frac{c_c}{K_c}\right)^2 - \left(\frac{c_n}{K_n}\right)^2}{1 + \left(\frac{c_c}{K_c}\right)^2 + \left(\frac{c_n}{K_n}\right)^2}. \tag{37}$$

Flux through the RyRs The flux from the jSR to the SL compartment is given by

$$J_s^{sl} = J_{\mathrm{RyR}} + J_{\mathrm{leak}}, \tag{38}$$

where J_{RyR} is the flux through the active RyR channels and J_{leak} is the flux through passive RyR channels that are always open, given by

$$J_{\mathrm{RyR}} = p \cdot r \cdot \alpha_{\mathrm{RyR}}(c_s - c_{sl}), \tag{39}$$

$$J_{\mathrm{leak}} = \gamma_{\mathrm{RyR}} \cdot \alpha_{\mathrm{RyR}}(c_s - c_{sl}), \tag{40}$$

respectively. Here, p represents the open probability of the active RyR channels and is given by

$$p = \frac{c_d^3}{c_d^3 + \kappa_{\mathrm{RyR}}^3}. \tag{41}$$

Furthermore, r is the fraction of RyR channels that are not inactivated and is governed by the equation

$$\frac{dr}{dt} = -\frac{J_{\mathrm{RyR}}}{\beta_{\mathrm{RyR}}} + \frac{\eta_{\mathrm{RyR}}}{p}(1 - r). \tag{42}$$

Passive diffusion fluxes between compartments The passive diffusion fluxes between intracellular compartments are given by

$$J_d^c = \alpha_d^c(c_d - c_c), \tag{43}$$

$$J_{sl}^c = \alpha_{sl}^c(c_{sl} - c_c), \tag{44}$$

$$J_n^s = \alpha_n^s(c_n - c_s). \tag{45}$$

Buffer fluxes The fluxes of free Ca^{2+} binding to a Ca^{2+} buffer are given by

$$J_d^b = V_d(k_{\mathrm{on}}^d c_d(B_{\mathrm{tot}}^d - b_d) - k_{\mathrm{off}}^d b_d), \tag{46}$$

$$J_{sl}^b = V_{sl}(k_{\mathrm{on}}^{sl} c_{sl}(B_{\mathrm{tot}}^{sl} - b_{sl}) - k_{\mathrm{off}}^{sl} b_{sl}), \tag{47}$$

$$J_c^b = V_c(k_{\mathrm{on}}^c c_c(B_{\mathrm{tot}}^c - b_c) - k_{\mathrm{off}}^c b_c), \tag{48}$$

$$J_s^b = V_s(k_{\mathrm{on}}^s c_s(B_{\mathrm{tot}}^s - b_s) - k_{\mathrm{off}}^s b_s). \tag{49}$$

Membrane fluxes The membrane Ca^{2+} fluxes, J_{CaL}, J_{bCa}, J_{pCa}, and J_{NaCa}, are given by

$$J_{\mathrm{CaL}} = -\frac{\chi C_m}{2F} I_{\mathrm{CaL}}, \qquad J_{\mathrm{pCa}} = -\frac{\chi C_m}{2F} I_{\mathrm{pCa}}, \tag{50}$$

$$J_{\mathrm{bCa}} = -\frac{\chi C_m}{2F} I_{\mathrm{bCa}}, \qquad J_{\mathrm{NaCa}} = \frac{\chi C_m}{F} I_{\mathrm{NaCa}}, \tag{51}$$

where I_{CaL}, I_{bCa}, I_{pCa}, and I_{NaCa} are defined by the expressions given above. Furthermore,

$$J_e^{sl} = J_{\mathrm{NaCa}} + J_{\mathrm{pCa}} + J_{\mathrm{bCa}}. \tag{52}$$

Intracellular Na^+ dynamics

The intracellular Na^+ concentration is governed by

$$\frac{d[\mathrm{Na}_i]}{dt} = -\frac{\chi C_m}{F}\left(I_{\mathrm{Na}} + I_{\mathrm{NaL}} + I_{\mathrm{bNa}} + 3I_{\mathrm{NaK}} + 3I_{\mathrm{NaCa}} + 0.3293 I_{\mathrm{f}}\right), \quad (53)$$

where the currents I_{Na}, I_{NaL}, I_{bNa}, I_{NaK}, I_{NaCa}, and I_{f} are specified above.

Protein regulation

The scaling factor n for the number of L-type calcium channels on the cell membrane is modeled by

$$\tau_n \frac{dn}{dt} = (c^* - c)H(c, n), \quad (54)$$

$$H(c, n) = h(n, n_-, \varepsilon_n)h(c, c^*, \varepsilon_c) + h(n_+, n, \varepsilon_n)h(c^*, c, \varepsilon_c), \quad (55)$$

$$h(a, b, \varepsilon) = \frac{1}{2}\left(1 + \tanh\left(\frac{a - b}{\varepsilon}\right)\right). \quad (56)$$

The function $H(c, n)$ is illustrated in Supplementary Figure 6.

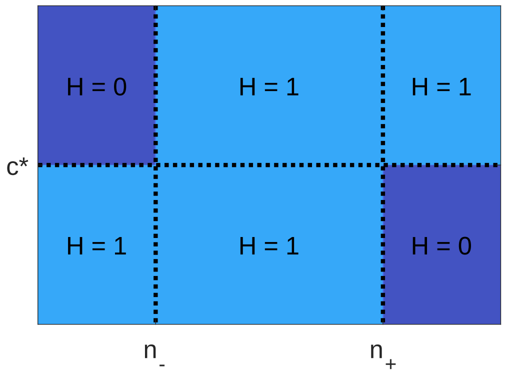

Supplementary Figure 6: Illustration of the function $H(c, n)$.

Nernst equilibrium potentials

The Nernst equilibrium potentials for the ion channels are defined as

$$E_{\mathrm{Na}} = \frac{RT}{F} \log\left(\frac{[\mathrm{Na}^+]_\mathrm{e}}{[\mathrm{Na}^+]_\mathrm{i}}\right), \tag{57}$$

$$E_{\mathrm{Ca}} = \frac{RT}{2F} \log\left(\frac{[\mathrm{Ca}^{2+}]_\mathrm{e}}{c_{sl}}\right), \tag{58}$$

$$E_{\mathrm{K}} = \frac{RT}{F} \log\left(\frac{[\mathrm{K}^+]_\mathrm{e}}{[\mathrm{K}^+]_\mathrm{i}}\right), \tag{59}$$

$$E_{\mathrm{Ks}} = \frac{RT}{F} \log\left(\frac{[\mathrm{K}^+]_\mathrm{e} + 0.018[\mathrm{Na}^+]_\mathrm{e}}{[\mathrm{K}^+]_\mathrm{i} + 0.018[\mathrm{Na}^+]_\mathrm{i}}\right), \tag{60}$$

$$E_{\mathrm{Cl}} = \frac{RT}{F} \log\left(\frac{[\mathrm{Cl}^+]_\mathrm{e}}{[\mathrm{Cl}^+]_\mathrm{i}}\right), \tag{61}$$

$$E_{\mathrm{f}} = -17 \text{ mV}, \tag{62}$$

for the parameter values given in Supplementary Table 2.

Baseline parameter values

Parameter	Description	Value
V_d	Volume fraction of the dyadic subspace	0.001
V_{sl}	Volume fraction of the SL compartment	0.028
V_c	Volume fraction of the bulk cytosol	0.917
V_s	Volume fraction of the jSR	0.004
V_n	Volume fraction of the nSR	0.05
χ	Cell surface to volume ratio	0.6 μm^{-1}

Supplementary Table 1: Default geometry parameters of the membrane model.

Parameter	Description	Value
C_m	Specific membrane capacitance	0.01 pF/μm^2
F	Faraday's constant	96.485 C/mmol
R	Universal gas constant	8.314 J/(mol·K)
T	Temperature	310 K
$[Ca^{2+}]_e$	Extracellular Ca^{2+} concentration	1.8 mM
$[Na^+]_e$	Extracellular sodium concentration	140 mM
$[K^+]_e$	Extracellular potassium concentration	5.4 mM
$[K^+]_e^b$	Baseline extracellular potassium concentration	5.4 mM
$[K^+]_i$	Intracellular potassium concentration	120 mM
$[Cl^-]_e$	Extracellular chloride concentration	150 mM
$[Cl^-]_i$	Intracellular chloride concentration	15 mM

Supplementary Table 2: Physical constants and ionic concentrations of the membrane model.

Parameter	Value	Parameter	Value
g_{Na}	7.056 mS/μF	$\bar{I}_{\text{NaCa}}$	2.22 μA/μF
g_{NaL}	0.021 mS/μF	$\bar{I}_{\text{pCa}}$	0.068 μA/μF
g_{to}	0.55 mS/μF	$\bar{J}_{\text{SERCA}}$	0.00024 mM/ms
g_{Kr}	1.2 mS/μF	α_{RyR}	0.0075 ms^{-1}
g_{Ks}	0.21 mS/μF	β_{RyR}	0.038 mM
g_{K1}	2.62 mS/μF	α_d^c	0.0034 ms^{-1}
g_{f}	0.0001 mS/μF	α_{sl}^c	0.15 ms^{-1}
g_{bCl}	0.0114 mS/μF	α_n^s	0.012 ms^{-1}
g_{CaL}	0.049 nL/(μF ms)	B_{tot}^c	0.07 mM
g_{bCa}	0.000832 mS/μF	B_{tot}^d	1.2 mM
g_{bNa}	0.00185 mS/μF	B_{tot}^{sl}	0.9 mM
g_{Kur}	2.475 mS/μF	B_{tot}^s	27 mM
$\bar{I}_{\text{NaK}}$	2.76 μA/μF		

Supplementary Table 3: Conductances and similar cell-specific parameter values in the membrane model formulation. Note that the parameter values of this table define the human left atrial version of the membrane model. For the human pulmonary vein version, the adjustment factors of Supplementary Table 4 are applied.

Parameter	PV scaling factor
g_{K1}	0.58
g_{Kr}	1.5
g_{Ks}	1.6
g_{to}	0.75
g_{CaL}	0.7

Supplementary Table 4: Adjustment factors for the pulmonary vein (PV) version of the membrane model, based on [13].

Parameter	Flux	Value
K_c	J_c^n	0.00025 mM
K_n	J_c^n	1.7 mM
γ_{RyR}	J_s^{sl}	0.001
κ_{RyR}	J_{RyR}	0.015 mM
η_{RyR}	J_s^{sl}	0.00001 ms^{-1}

Supplementary Table 5: Parameters for the intracellular Ca^{2+} fluxes of the membrane model.

Parameter	Current	Value
k_{sat}	I_{NaCa}	0.3
ν	I_{NaCa}	0.3
K_{act}	I_{NaCa}	0.00015 mM
$K_{\mathrm{Ca},i}$	I_{NaCa}	0.0036 mM
$K_{\mathrm{Ca},e}$	I_{NaCa}	1.3 mM
$K_{\mathrm{Na},i}$	I_{NaCa}	12.3 mM
$K_{\mathrm{Na},e}$	I_{NaCa}	87.5 mM
$K_{\mathrm{Na},i}^{\mathrm{NaK}}$	I_{NaK}	$(11\ \mathrm{mM})\cdot(Q_{10}^{\mathrm{KNaK}})^{Q^p}$
$K_{\mathrm{K},e}$	I_{NaK}	1.5 mM
K_{pCa}	I_{pCa}	0.0005 mM

Supplementary Table 6: Additional parameters for the membrane currents of the membrane model.

Parameter	Compartment	Value
k_{on}^{c}	Bulk cytosol	40 $\text{ms}^{-1}\text{mM}^{-1}$
k_{off}^{c}	Bulk cytosol	0.03 ms^{-1}
k_{on}^{d}	Dyad	100 $\text{ms}^{-1}\text{mM}^{-1}$
k_{off}^{d}	Dyad	1 ms^{-1}
k_{on}^{sl}	Subsarcolemmal space	100 $\text{ms}^{-1}\text{mM}^{-1}$
k_{off}^{sl}	Subsarcolemmal space	0.15 ms^{-1}
k_{on}^{s}	Junctional SR	100 $\text{ms}^{-1}\text{mM}^{-1}$
k_{off}^{s}	Junctional SR	65 ms^{-1}

Supplementary Table 7: Transition rates for the Ca^{2+} buffers of the membrane model.

$\alpha_{\text{Kr},1}$	$0.4 \cdot T_* \cdot e^{24.335+T_*^{-1}(0.0112v-25.914)}$
$\beta_{\text{Kr},1}$	$T_* \cdot e^{13.688+T_*^{-1}(-0.0603v-15.707)}$
$\alpha_{\text{Kr},2}$	$0.4 \cdot T_* \cdot e^{22.746+T_*^{-1}(-25.914)}$
$\beta_{\text{Kr},2}$	$T_* \cdot e^{13.193+T_*^{-1}(-15.707)}$
$\alpha_{\text{Kr},3}$	$0.4 \cdot T_* \cdot e^{22.098+T_*^{-1}(0.0365v-25.914)}$
$\beta_{\text{Kr},3}$	$T_* \cdot e^{7.313+T_*^{-1}(-0.0399v-15.707)}$
$\alpha_{\text{Kr},4}$	$T_* \cdot \left(\frac{[\text{K}^+]_e^b}{[\text{K}^+]_e}\right)^{0.4} e^{30.016+T_*^{-1}(0.0223v-30.888)}$
$\beta_{\text{Kr},4}$	$T_* \cdot e^{30.061+T_*^{-1}(-0.0312v-33.243)}$

Supplementary Table 8: Transition rates for the I_{Kr} Markov model. Here, $T_* = \frac{T}{310\text{ K}}$, where T is the temperature.

Parameter	Value	Parameter	Value
n_-	0.28	n_+	3
ε_n	0.01	ε_c	10^{-7} mM
c^* (LA)	$2.22{\cdot}10^{-4}$ mM	c^* (PV)	$1.79{\cdot}10^{-4}$ mM
τ_n	30,000 mMms		

Supplementary Table 9: Parameters for the regulation of the number of L-type calcium channels in the cell membrane.

Current	Gate	z_∞	α_z	β_z	τ_z
I_{Na}	m	$\frac{1}{(1+e^{(-57-v)/9})^2}$	$0.13e^{-((v+46)/16)^2}$	$0.06e^{-((v-5)/51)^2}$	$\frac{\alpha_m+\beta_m}{(Q_{10}^{\text{Na}})^{Q_p}}$
	j	$\frac{1}{(1+e^{(v+72)/7})^2}$	0, if $v \geq -40$<br>$\frac{\begin{pmatrix}-2.5\cdot 10^4 e^{0.2v} \\ -7\cdot 10^{-6}e^{-0.04v}\end{pmatrix}(v+38)}{1+e^{0.3(v+79)}}$, otherwise	$\frac{0.6e^{0.06v}}{1+e^{-0.1(v+32)}}$, if $v \geq -40$<br>$\frac{0.02e^{-0.01v}}{1+e^{-0.14(v+40)}}$, otherwise	$\frac{1}{(\alpha_j+\beta_j)(Q_{10}^{\text{Na}})^{Q_p}}$
I_{NaL}	m_L	$\frac{1}{1+e^{(-43-v)/5}}$	$\frac{1}{6.8e^{(v+12)/35}}$	$8.6e^{-(v+77)/6}$	$\frac{\alpha_m+\beta_m}{(Q_{10}^{\text{NaL}})^{Q_p}}$
	h_L	$\frac{1}{1+e^{(v+88)/7.5}}$			$\frac{200\text{ ms}}{(Q_{10}^{\text{NaL}})^{Q_p}}$
I_{CaL}	d	$\frac{1}{1+e^{-(v+20)/6}}$	$\frac{1-e^{-\frac{v+5}{6}}}{0.035(v+5)}$		$\alpha_d d_\infty$
	f	$\frac{1}{1+e^{(v+35)/9}}+\frac{0.6}{1+e^{(50-v)/20}}$	$\frac{1}{0.02e^{-(0.034(v+14.5)^2)}+0.02}$		α_f
	f_{Ca}	$\frac{1.7c_d}{1.7c_d+0.012}$	$\frac{1}{1.7c_d^{1.5}+0.012}$		α_{Ca}
I_{to}	q_{to}	$\frac{1}{1+e^{(v+53)/13}}$	$\frac{39}{0.57e^{-0.08(v+44)}+0.065e^{0.1(v+46)}}$	6	$\frac{\alpha_{q_{\text{to}}}+\beta_{q_{\text{to}}}}{(Q_{10}^{\text{to}})^{Q_p}}$
	r_{to}	$\frac{1}{1+e^{-(v-22.3)/18.75}}$	$\frac{14.4}{e^{0.09(v+30.61)}+0.37e^{-0.12(v+24)}}$	2.75	$\frac{\alpha_{r_{\text{to}}}+\beta_{r_{\text{to}}}}{(Q_{10}^{\text{to}})^{Q_p}}$
I_{Ks}	x_{Ks}	$\frac{1}{1+e^{-(v+3.8)/14}}$	$\frac{990}{1+e^{-(v+2.4)/14}}$		$\frac{\alpha_{x_{\text{Ks}}}}{(Q_{10}^{\text{Ks}})^{Q_p}}$
I_{Kur}	x_{Kur1}	$\frac{1}{1+e^{-(v+30.3)/9.6}}$	$\frac{0.65}{e^{-(v+10)/8.5}+e^{-(v-30)/59}}$	$\frac{0.65}{2.5+e^{(v+82)/17}}$	$\frac{1}{(\alpha_{x_{\text{Kr1}}}+\beta_{x_{\text{Kr1}}})(Q_{10}^{\text{Kur}})^{Q_p}}$
	x_{Kur2}	$\frac{1}{1+e^{(v-99.45)/27.48}}$	$\frac{1}{21+e^{-(v-185)/28}}$	$e^{(v-158)/16}$	$\frac{1}{(\alpha_{x_{\text{Kr2}}}+\beta_{x_{\text{Kr2}}})(Q_{10}^{\text{Kur}})^{Q_p}}$
I_{f}	x_{f}	$\frac{1}{1+e^{(v+78)/5}}$	$\frac{1900}{1+e^{(v+15)/10}}$		$\frac{\alpha_{x_{\text{f}}}}{(Q_{10}^{\text{f}})^{Q_p}}$

Supplementary Table 10: Specification of the parameters z_∞ and τ_z, for $z = m$, j, m_L, h_L, d, f, f_{Ca}, q_{to}, r_{to}, x_{Ks}, x_{Kur1}, x_{Kur2}, and x_{f} in the equations for the gating variables (2).

Supplementary References

[1] Karoline H Jæger and Aslak Tveito. Efficient, cell-based simulations of cardiac electrophysiology; the Kirchhoff Network Model (KNM). *NPJ Systems Biology and Applications*, 9(1):25, 2023.

[2] Karoline H Jæger and Aslak Tveito. The simplified Kirchhoff network model (SKNM): a cell-based reaction–diffusion model of excitable tissue. *Scientific Reports*, 13:16434, 2023.

[3] Karoline H Jæger, Andrew G Edwards, Wayne R Giles, and Aslak Tveito. Arrhythmogenic influence of mutations in a myocyte-based computational model of the pulmonary vein sleeve. *Scientific Reports*, 12(1):1–18, 2022.

[4] Eleonora Grandi, Francesco S Pasqualini, and Donald M Bers. A novel computational model of the human ventricular action potential and Ca transient. *Journal of Molecular and Cellular Cardiology*, 48(1):112–121, 2010.

[5] Thomas O'Hara, László Virág, András Varró, and Yoram Rudy. Simulation of the undiseased human cardiac ventricular action potential: Model formulation and experimental validation. *PLoS Computational Biology*, 7(5):e1002061, 2011.

[6] Michelangelo Paci, Jari Hyttinen, Katriina Aalto-Setälä, and Stefano Severi. Computational models of ventricular-and atrial-like human induced pluripotent stem cell derived cardiomyocytes. *Annals of Biomedical Engineering*, 41(11):2334–2348, 2013.

[7] Martin Fink, Denis Noble, Laszlo Virag, Andras Varro, and Wayne R Giles. Contributions of HERG K^+ current to repolarization of the human ventricular action potential. *Progress in Biophysics and Molecular Biology*, 96(1-3):357–376, 2008.

[8] Mark J McPate, Rona S Duncan, James T Milnes, Harry J Witchel, and Jules C Hancox. The N588K-HERG K^+ channel mutation in the 'short QT syndrome': mechanism of gain-in-function determined at 37 °C. *Biochemical and Biophysical Research Communications*, 334(2):441–449, 2005.

[9] Makarand Deo, Yanfei Ruan, Sandeep V Pandit, Kushal Shah, Omer Berenfeld, Andrew Blaufox, Marina Cerrone, Sami F Noujaim, Marco

Denegri, José Jalife, and Silvia G Priori. KCNJ2 mutation in short QT syndrome 3 results in atrial fibrillation and ventricular proarrhythmia. *Proceedings of the National Academy of Sciences*, 110(11):4291–4296, 2013.

[10] Eleonora Grandi, Sandeep V Pandit, Niels Voigt, Antony J Workman, Dobromir Dobrev, José Jalife, and Donald M Bers. Human atrial action potential and Ca^{2+} model: sinus rhythm and chronic atrial fibrillation. *Circulation Research*, 109(9):1055–1066, 2011.

[11] Marc Courtemanche, Rafael J Ramirez, and Stanley Nattel. Ionic mechanisms underlying human atrial action potential properties: insights from a mathematical model. *American Journal of Physiology-Heart and Circulatory Physiology*, 275(1):H301–H321, 1998.

[12] Ingrid E Christophersen, Morten S Olesen, Bo Liang, Martin N Andersen, Anders P Larsen, Jonas B Nielsen, Stig Haunsø, Søren-Peter Olesen, Arnljot Tveit, Jesper H Svendsen, and Nicole Schmitt. Genetic variation in KCNA5: impact on the atrial-specific potassium current I_{Kur} in patients with lone atrial fibrillation. *European Heart Journal*, 34(20):1517–1525, 2013.

[13] Joachim R Ehrlich, Tae-Joon Cha, Liming Zhang, Denis Chartier, Peter Melnyk, Stefan H Hohnloser, and Stanley Nattel. Cellular electrophysiology of canine pulmonary vein cardiomyocytes: action potential and ionic current properties. *The Journal of Physiology*, 551(3):801–813, 2003.
